# Determining the Role of the Aromatic Ring of *N*-Arylmethyl *ent*-conduramine F-1 in their Interactions with α-Glucosidases by Saturation Transfer Difference NMR Spectroscopy Experiments

**DOI:** 10.1002/open.201100004

**Published:** 2012-01-02

**Authors:** Antonio Hernández Daranas, Sonia Koteich Khatib, Robert Lysek, Pierre Vogel, José A Gavín

**Affiliations:** [a] Instituto Universitario de Bio-Orgánica “Antonio González”, University of La LagunaAv. Fco. Sánchez 2, 38206 La Laguna, Tenerife (Spain) E-mail: adaranas@ull.esjgavin@ull.es; [b] Department. of Chemistry, Faculty of Sciences, University of los Andes, Campus Universitario “Pedro Rincón Gutiérrez”5101 Mérida (Venezuela); [c] Laboratory of Glycochemistry and Asymmetric Synthesis, Swiss Federal Institute of Technology (EPFL)Batochime, 1015 Lausanne-Dorigny (Switzerland)

**Keywords:** alpha-glucosidases, conduramines, hydrogen bonds, molecular modeling, saturation transfer difference NMR spectroscopy

Glucosidases are a group of enzymes responsible of the glycosidic bond cleavage with different specificities depending on the number of monosaccharides, the position of cleavage, and the configuration of the hydroxy groups in their substrates. In particular, inhibitors of α-1,4-glucosidases have generated great interest as potential therapeutic agents for the treatment of type II diabetes (e.g., miglitol (*N*-2-hydroxyethyl-1-deoxynojirimycin), Glyset, Diastabol, Glucobay),^1^, ^2^ obesity,^3^ hepatitis B and C^4^, ^5^ and other viral diseases,^6^ and cancer.^7^

Previously, we have shown that N-benzylation of (+)-*ent*-conduramine F-1 (**1**) significantly increases its inhibitory activity toward α-1,4-glucosidase from yeast.^8^ Because of their relatively high hydrophobicity, *N*-benzyl derivatives of **1** might represent α-1,4-glucosidase inhibitors with improved bioavailability and pharmacokinetics.^9^ So far, rational drug design has not been applied to inhibitors of α-1,4-glucosidases, because the structural information available is very scarce and only related to the free forms of the protein. The lack of structural information on the nature of the interactions between α-1,4-glucosidases and their inhibitors has made it a difficult task to discover good lead compounds. In this work, we have attempted to understand the role of the aryl moieties in improving the inhibitory activities of *N*-benzyl derivatives of **1** using saturation transfer difference (STD) NMR spectroscopy. The method allows the binding of a ligand to a receptor to be characterize by using small amounts of protein (micromolar range), and it is very useful for mapping the binding epitope of the ligand with atomic resolution. Ligand protons that are in close contact with the protein binding pocket experience a larger fraction of saturation transfer than protons further away.^10^ Thus, protons of ligand directly involved in binding show larger signal increments than other ligand protons in STD NMR spectra.

α-1,4-Glucosidase from *Saccharomyces cerevisiae* was used in this study as a model system to evaluate the interactions of ligand **1** and its *N*-benzyl derivatives **2**–**4** with the enzyme by STD NMR spectroscopy (Figure 1). Significant STD effects were observed for all ligands in the presence of α-1,4-glucosidase, which indicated that they are bound to the enzyme under the test conditions. In addition, to validate the stability of the inhibitors, ^1^H NMR spectra of the complexes were acquired after being left in solution for several days, and hydrolysis products were not observed.

**Figure 1:**
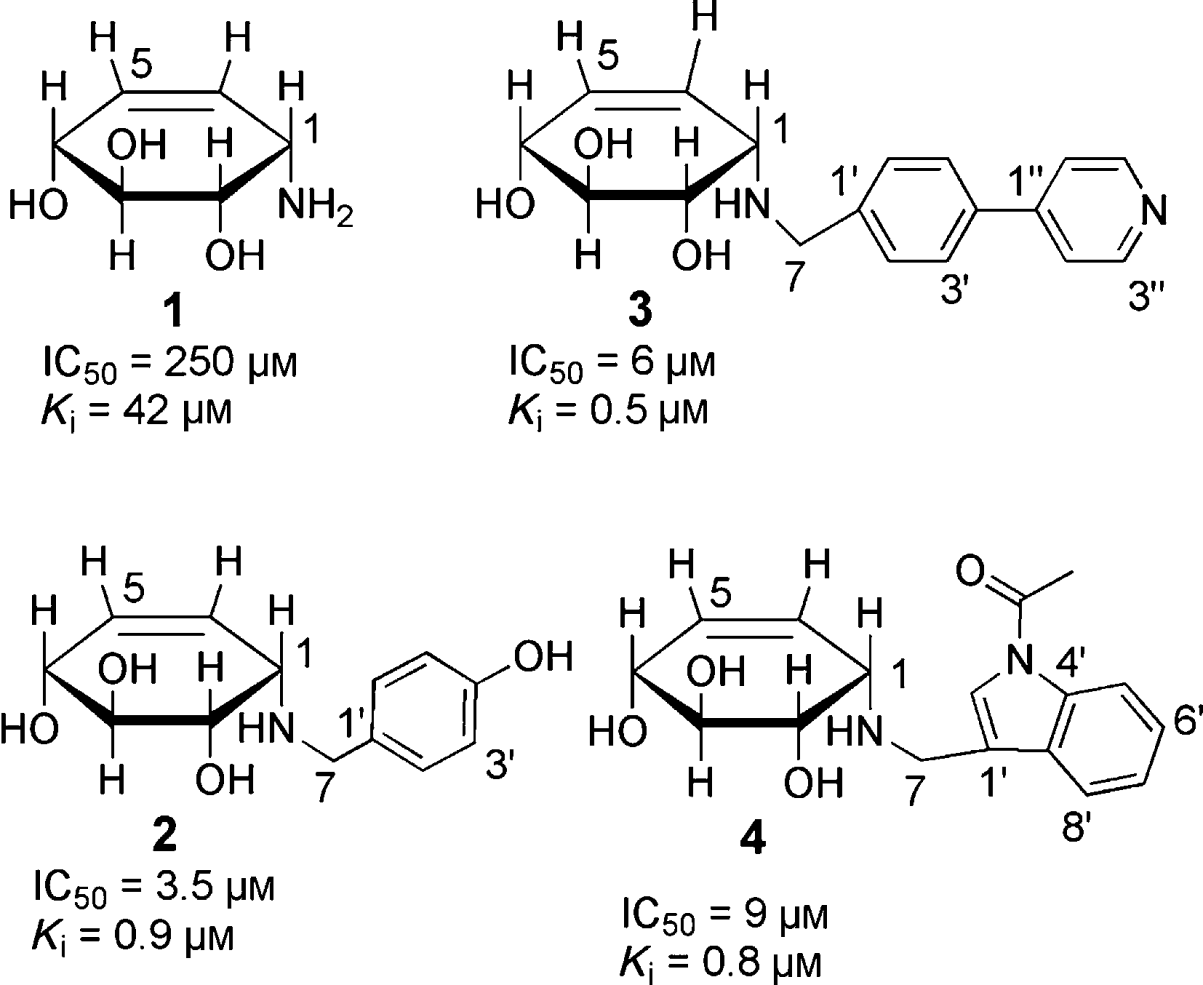
Chemical structures of compounds 1–4. The inhibitory activities are also shown as IC_50_ and *K*_i_ values.^8^

The size of the observed STD effect does not only depend on the proximity of the proton to the receptor. In fact, if the longitudinal relaxation times (*T*1) of individual ligand protons are quite different, there is a severe interference on the epitope map for ligand–receptor interactions derived from STD measurements.^11^ Therefore, it is essential to consider the importance of this circumstance on STD experiments performed for molecules that have protons with substantially different *T*1 values. Measurements of *T*1 values for individual ligand protons in complex with α-1,4-glucosidase were undertaken for all ligands (Table 1). In general, H4 and H5 showed longer *T*1 values in all inhibitors, and important relative differences were observed within each ligand, with the exception of **2** that showed *T*1 values of 0.4–0.6 s.

**Table 1:** Longitudinal relaxation times (*T*1) calculated for ligands 1–4 in complex with α-1,4-glucosidase.^[a]^

1	*T*1	2	*T*1	3	*T*1	4	*T*1
H1	0.93	H1/H2	0.42	H1/H2	0.77	H1/H2	0.88
H2	1.05	H3	0.52	H3	0.81	H3	1.17
H3	1.14	H4	0.57	H4	1.09	H4	1.52
H4	1.41	H5	0.60	H5	1.05	H5	1.25
H5	1.78	H6	0.53	H6	0.81	H6	0.87
H6	1.28	H7	0.29	H7	0.46	H2′	1.08
		H2′/H6′	0.58	H2′/H6′	0.86	H5′	1.99
		H3′/H5′	0.69	H3′/H5′	0.89	H6′	1.26
				H2′′/H6′′	0.77	H7′	1.27
				H3′′/H5′′	0.29	H8′	1.23
						N—Ac	4.70

[a] *T*1 values for individual protons measured for each inhibitor in complex with α-1,4-glucosidase are noted in seconds (s).

The use of saturation times shorter than *T*1 has been suggested for improving the accuracy of STD results. However, under these conditions, poor signal-to-noise ratios are usually obtained due to low magnetization transfer from the receptor.^11^ To overcome this problem, we decided to use STD initial growing rates (STD_0_), calculated from the fitting of the saturation time data to monoexponential Equation (1):


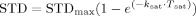
(1)

where STD stands for the STD signal intensity of a given proton at saturation time *T*_sat_, STD_max_ is the maximal STD intensity obtainable, and *k*_sat_ stands for the observed saturation rate constant.^12^ Therefore, STD data were acquired on samples containing each ligand in the presence of α-1,4-glucosidase (200:1 molar ratio) at a series of saturation times (*T*_sat_=0.25, 0.5, 1, 2, 3, 5 s), as shown in Figure 2 a. As a representative example, STD build-up curves for ligand **3** can be seen in Figure 2 b.

**Figure 2:**
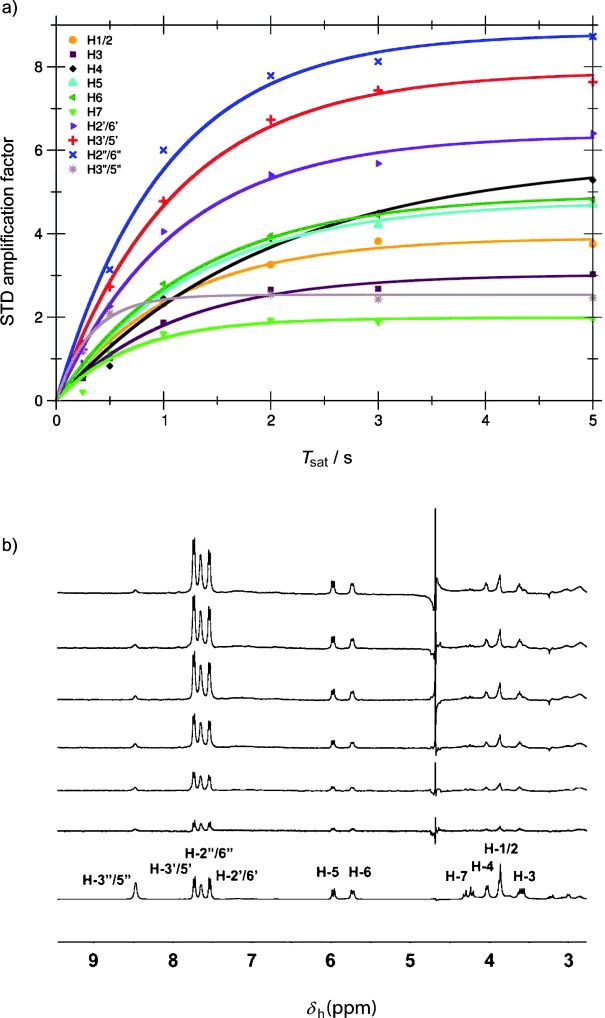
a) STD build-up curves for all protons in ligand 3. Experimental data were fitted to a rising exponential to calculate *K*_sat_ and STD. b) Reference NMR spectrum of a mixture of compound 3 and 0.15 mm of α-1,4-glucosidase in a 200:1 molar ratio. STD NMR spectra obtained at increasing saturation times of 0.25, 0.5, 1, 2, 3, and 5 s (from bottom to top).

Clearly, the signal enhancement for the protons with shorter *T*1 values (0.29 s for H3′′ and H5′′) have already reached a plateau after 1 s. A slightly longer time was observed for H7 that showed the second shorter *T*1 value (0.46 s). Conversely, the other protons still developed signal up to saturation times around 2 or 3 s depending on their *T*1 values (longer than 0.8 s in all cases). Similar results were obtained for the other ligands.

We believe that when comparing ligands with similar structures that bind into the same binding site, the important point is to focus on the overall pattern, and but not on the individual values of each proton. For example, for inhibitors 2–4, the STD enhancements are larger for the aromatic protons than for the conduramine protons (Table 2). In addition, it should be noted that the relative differences observed are bigger for the ligand with a tryptophan moiety (4), followed by the ligand with a pyridine ring (3), and finally by the phenolic derivative (2), for which the differences are not so important. If the purpose of N-benzylation of 1 was to add a substituent that would simply expel water molecules from the α-1,4-glucosidase active site and thus increase the binding constant^13^, ^14^ because of more a favorable binding entropy,^15^ we would expect the opposite behavior: larger STD effects at the H1 to H4 positions due to specific interactions (hydrogen bond) between the conduramine and the enzyme.^16^ Thus, the role of the N-substituents in 2–4 should be to interact directly with the protein. We also observed that the conduramine protons are affected by N-benzylation, independent of the nature of the aromatic ring of the inhibitor following the same general trend: H1,2,3 always show the smallest STD effect, whereas the olefinic protons H5,6 show a slight enhancement of the signal intensity in 2–4 compared with the olefinic signals of 1.

**Table 2:** Saturation transfer difference (STD) effects calculated for ligands 1–4 in complex with α-1,4-glucosidase.^[a]^

1	STD_fit_	2	STD_fit_	3	STD_fit_	4	STD_fit_
H1	98	H1/H2	79	H1/H2	124	H1/H2	143
H2	41	H3	54	H3	94	H3	71
H3	64	H4	100	H4	100	H4	100
H4	100	H5	97	H5	127	H5	143
H5	71	H6	101	H6	133	H6	200
H6	65	H7	110	H7	97	H2′	386
		H2′/H6′	121	H2′/H6′	200	H5′	1029
		H3′/H5′	147	H3′/H5′	245	H6′	1429
				H2′′/H6′′	303	H7′	643
				H3′′/H5′′	273	H8′	471
						N—Ac	29

[a] STD_fit_ corresponds to the relative STD intensity for each ligand calculated from fitting the data to monoexponential Equation (1).

The enzyme used in our NMR spectroscopy experiments was α-1,4-glucosidase derived from the yeast, *S. cerevisiae*. Unfortunately, although it is well known that the *MAL 12* gene regulates the expression of this enzyme, as yet, no experimental 3D information is available. However, a number of homologous sequences with 3D structures deposited at the Protein Data Bank (PDB) are available and were found by using a basic local alignment search tool program (BLASTP) search.^17^ Of these sequences, the oligo-1,6-glucosidases from *S. cerevisiae* (PDB: 3AJ7)^18^ and *Bacillus cereus* (PDB: 1UOK)^19^ showed the highest sequence identity (72 % and 38.5 %, respectively) compared with the sequence of α-1,4-glucosidase from *S. cerevisiae* (see Supporting Information). Therefore, taking into account the good sequence identity between the α-1,4-glucosidase and the oligo-1,6-glucosidase from *S. cerevisiae*, we decided to build a theoretical model of α-1,4-glucosidase using homology modeling techniques.^20^ In fact, in α-1,4-glucosidase, the catalytic triad D 215, E 277, D 352 characteristic of oligo-1,6-glucosidase is conserved (D 211, E 273, D 346 in α-1,4-glucosidase) together with the residues D 69, H 112, R 213, H 351, and R 442 (D 65, H 108, R 209, H 345, and R 436 in α-1,4-glucosidase), which have been found to participate in a hydrogen-bond network within the active site of oligo-1,6-glucosidase.^18^

Molecular docking simulations were then undertaken to gain further insight into the most probable binding mode of the studied ligands.^21^ Considering that the residues within the active site of several members of the glycoside hydrolase family are highly conserved, we defined the binding site of α-1,4-glucosidase around those residues. To validate our results, the docked conformations for each ligand were superimposed and compared with the crystal structure of oligo-1,6-glucosidase in complex with maltose (PDB: 3A4A).^18^ The simulation results predict that all ligands interact with the active site residues in a very similar way, which suggests that the docking protocol was reasonable in identifying the binding conformation accurately.

The four ligands used in this study share the *ent*-conduramine moiety, although ligands **2**–**4** also include different aromatic moieties. The best docking solutions for all ligands show almost superimposable poses for their respective *ent*-conduramine moieties, although the aromatic rings are accommodated in slightly different positions. Therefore, as otherwise stated, our discussion is common to all ligands. A visual assessment and a ligplot^22^ analysis of the results suggested that an extensive hydrogen-bond network could be formed between the *ent*-conduramine and several residues within the active site. In fact, R 436 can form a hydrogen bond with the hydroxy groups at C2 and C4 (2.75–2.85 Å). In addition, the hydroxy group at C4 is also in a good position to form a hydrogen bond with D 65 (2.7 Å). The hydroxy group at C3 could form hydrogen bonds with the catalytic residues D 211 (2.9 Å) and D 346 (3.0–3.2 Å), or even with H 345, although this residue is located slightly further away (3.3–3.4 Å). Likewise, the amine group at C1 could be involved in a hydrogen bond with T 212, and the double bond at C5–C6 could potentially interact positively with F 174. With regard to the aromatic moieties of the studied ligands, our simulations predict that although the binding cavity is narrow, there is enough space to accommodate them. In fact, the aromatic rings of ligands **2** and **4** could be stabilized by several hydrophobic contacts with F 155 and A 275, and our model suggests they would essentially be sandwiched by F 154 and F 297, with each residue interacting with a different face of the ligand. For inhibitor **3**, in addition to the previously mentioned contacts, modeling also predicts interactions with H 236 and H 276 due to its bigger size (see Figure 3 and the Supporting Information).

**Figure 3:**
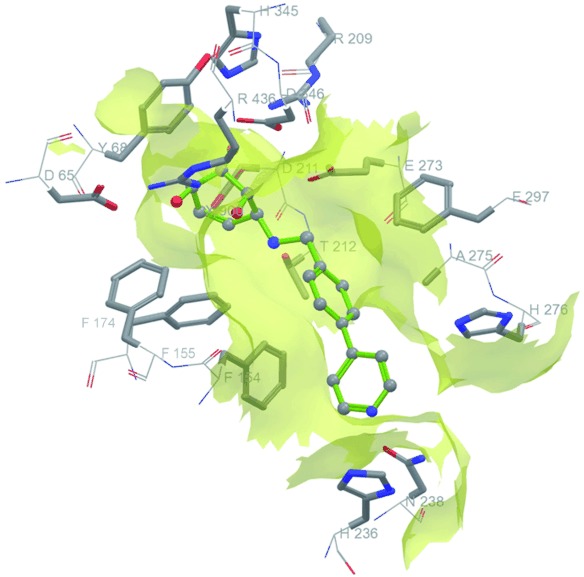
Docked conformation obtained for ligand **3** in the modeled α-1,4-glucosidase active site. Only those residues within a distance of 4 Å of the ligand are shown. The molecular surface of the binding cavity is shown in yellow. The ligand is depicted with green bonds for clarity.

In conclusion, introduction of aromatic moieties on iminosugars and their analogues might decrease^24^, ^25^ or increase^26^ their inhibitory activity against different glycosidases. In the case of (+)-*ent*-conduramine F-1 (**1**), which is a moderate inhibitor of α-1,4-glucosidases, N-benzylation gives a derivative with significantly increased inhibitory activity. By applying STD NMR spectroscopy, we have demonstrated for the first time that the aromatic moieties of *N*-benzyl *ent*-conduramine F-1 derivatives interact strongly with α-1,4-glucosidase from *S. cerevisiae*—more strongly than the conduramine moiety. Interestingly, upon N-benzylation of **1**, the olefinic protons experience enhanced interaction with the enzyme, but this is not true for the other protons in the ligand.

## Supporting Information

Detailed facts of importance to specialist readers are published as ”Supporting Information”. Such documents are peer-reviewed, but not copy-edited or typeset. They are made available as submitted by the authors.
