# Supplementary material for: Determining the Role of the Aromatic Ring of N-Arylmethyl ent-conduramine F-1 in their Interactions with α-Glucosidases by Saturation Transfer Difference NMR Spectroscopy Experiments
Source: ChemistryOpen. 2012 Jan 2;1(1):13–6. doi: 10.1002/open.201100004 (PMC3922434; doi:10.1002/open.201100004)
Supplement: Supplementary file 1 [file open0001-0013-SD1.pdf]

ChemistryOPEN

Supporting Information

© Copyright Wiley-VCH Verlag GmbH & Co. KGaA, 69451 Weinheim, 2012

Determining the Role of the Aromatic Ring of *N*-Arylmethyl *ent*-conduramine F-1 in their Interactions with α-Glucosidases by Saturation Transfer Difference NMR Spectroscopy Experiments

Antonio Hernández Daranas,*[a] Sonia Koteich Khatib,[a, b] Robert Lysek,[c] Pierre Vogel,[c] and José A. Gavín*[a]

open_201100004_sm_miscellaneous_information.pdf

Experimental section

NMR sample preparation: Samples were prepared in 75 mM Na_3PO_4, 25 mM citric acid (pH* 7.0) buffer solution, in D2O (99.99 %). Baker's yeast α-1,4-glucosidase (E.C. 3.2.1.20) was purchased from Sigma-Aldrich and used without further purification. The samples in presence of α-1,4-glucosidase were prepared using an inhibitor/enzyme molar ratio of 200:1 (0.15 mM of α-1,4-glucosidase).

NMR experiments: NMR experiments were recorded in an AVANCE Bruker instrument operating at 400.13 MHz. STD experiments were performed at 298 K, using Watergate solvent suppression, at 0.25, 0.5, 1.0, 2.0, 3.0 and 5.0 s saturation times using a train of Gaussian shaped pulses of 48 ms with an excitation bandwidth of 140 Hz, spaced by 1.0 ms delays. Total recycle delay was set to a fixed value of 5.0 s. A 10 ms $T_{1\rho}$ filter ($gB_1/2\pi$ = 6000 Hz) was used to remove residual protein signals. On-resonance irradiation was performed at 0 ppm and off-resonance irradiation was at 30.0 ppm; appropriate blank experiments were also performed to assure the absence of direct irradiation on the ligand. T1 measurements on the 200:1 inhibitor/enzyme samples were acquired with the inversion recovery pulse sequence using 0.1, 0.2, 0.4, 0.8, 1.0, 2.0, 4.0 and 8.0 s delays.

Homology modeling: The sequence of Baker's yeast α-1,4-glucosidase (E.C. 3.2.1.20) as obtained from the MAL12 gene (UniprotKB/Swiss-Prot database access number P53341) consists of a single polypeptide chain of 584 amino acids. This sequence was used to search within the PDB repository, finding the crystallographic structures of *Saccharomyces cereviciae* oligo-1,6-glucosidase (PDB code 3AJ7) and in complex with maltose (PDB code 3A4A) as well as oligo-1,6-glucosidase from *Bacillus cereus* (PDB code 1UOK) with a sequence identity of 72% and 38% respectively. The model was built using the Swiss-Model server [1] and the generated structure checked for their overall structural quality using the Pro-Check program. [2] 99% of the residues were found within the allowed backbone conformations, according to the Ramachandran plot.

Only 3 residues (0.6%) lay in generously allowed regions and just two residues (0.4%) had a disallowed conformation.

Docking: The AutoDockVina software package [3] was used to dock compounds **1** to **4** to the catalytic site of the modeled structure of α-1,4-glucosidase. The docking area was set around the active site within a box of 22 Å x 23 Å x 25 Å that was centered around the binding position occupied by maltose in the crystallographic structure of oligo-1,6-glucosidase from *S. cerevisiae* (PDB code 3A4A). An exhaustiveness parameter of 10 was used for the search.

[1] A) K. Arnold, L. Bordoli, J. Kopp, T. Schwede, *Bioinformatics*, **2006**, 22, 195-201. B) F. Kiefer, K. Arnold, M. Künzil, L. Bordoli, T. Schwede. *Nucleic Acids Research*, **2009**, 37, D387-D392.

[2] R. Luthy, J.U. Bowie, D. Eisenberg, *Nature*, **1992**, *356*, 83-85.

[3] O. Trott, A.J. Olson, *J. Comp. Chem*, **2010**, *31*, 455-461.

NMR data for compound 1: (+)-(1S,2S,3S,4R)-1-aminociclohex-5-en-2,3,4-triol.

^{1}H NMR (400MHz, D_2O): δ (ppm), *J* (Hz); 5.9 (H-5, d, $J_{5,6}$ = 10.1); 5.7 (H-6, d, $J_{6,5}$ = 10.1); 4.0 (H-4, d, $J_{4,3}$ = 9.4); 3.8 (H-1 and H-2, m); 3.6 (H-3, t, *J* = 9.4).

^{13}C NMR (100MHz, D_2O): δ (ppm) 134.7 (C-5); 122.5 (C-6); 71.6 (C-3); 71.3 (C-4); 67.5 (C-2); 49.7 (C-1).

NMR data for compound 2: (+)-(1S,2S,3S,4R)-1-[(4hidroxibencil)amino]ciclohex-5-en-2,3,4-triol.

^{1}H NMR (400MHz, D_2O): δ (ppm), *J* (Hz); 7.3 (H-2' and H-6', d, *J* = 9.2); 6.8 (H-3' and H-5', d, *J* = 9.2); 5.9 (H-5, d, $J_{5,6}$ = 11.6); 5.6 (H-6, d, $J_{6,5}$ = 11.6); 4.2 (H-7a, d, $J_{7a,b}$ = 13.4); 4.1 (H-7b, d, $J_{7b,a}$ = 13.4); 4.0 (H-4, d, $J_{4,3}$ = 7.8); 3.8 (H-1 and H-2, m); 3.6 (H-3, t, *J* = 7.8).

^{13}C NMR (100MHz, D_2O): δ (ppm) 157.3 (C-1'); 136.6 (C-4'); 131.7 (C-2' and C-6'); 122.1 (C-5); 119.4 (C-6); 116.1 (C-3' and C-5'); 71.7 (C-3); 70.6 (C-4); 66.6 (C-2); 54.7 (C-1); 48.7 (C-7).

NMR data for compound 3: (+)-(1S,2S,3S,4R)-1-[(4-piridin-4-il-bencil)amino]ciclohex-5-en-2,3,4-triol.

^{1}H NMR (400MHz, D_2O): δ (ppm), *J* (Hz); 8.5 (H-3" and H-5", s broad); 7.7 (H-3' and H-5', d, *J* = 7.9); 7.6 (H-2" and H-6", s broad); 7.5 (H-2' and H-6', d, *J* = 7.9); 5.9 (H-5, d, $J_{5,6}$ = 10.9); 5.7 (H-6, d, $J_{6,5}$ = 10.9); 4.3 (H-7a, d, $J_{7a,b}$ = 13.2); 4.2 (H-7b, d, $J_{7b,a}$ = 13.2); 4.1 (H-4, d, $J_{4,3}$ = 8.1); 3.9 (H-1 and H-2, m); 3.6 (H-3, t, *J* = 8.1).

^{13}C NMR (100MHz, D_2O): δ (ppm) 148.7 (C-3" and C-5"); 148.7 (C-1"); 138.4 (C-4'); 136.4 (C-5); 132.4 (C-1'); 130.5 (C-2' and C-6'); 127.8 (C-3' and C-5'); 122.1 (C-2" and C-6"); 119.9 (C-6); 71.9 (C-3); 70.7 (C-4); 66.9 (C-2); 55.3 (C-1); 48.9 (C-7).

NMR data for compound 4: (+)-(1S,2S,3S,4R)-1-{[(1-acetil-1*H*-indol-3-il)]metil}amino}ciclohex-5-en-2,3,4-triol.

^{1}H NMR (400MHz, D_2O): δ (ppm), *J* (Hz); 8.1 (H-5', d, $J_{5',6'}$ = 8.1); 7.7 (H-2', s); 7.5 (H-8', d, $J_{7',8'}$ = 8.1); 7.3 (H-6', dd, $J_{6',5'}$ = 8.1 $J_{6',7'}$ = 8.1); 7.2 (H-7', dd, $J_{7',6'}$ = 8.1 $J_{7',5'}$ = 8.1); 5.9 (H-5, d, $J_{5,6}$ = 10.7); 5.7 (H-6, d, $J_{6,5}$ = 10.7); 4.3 (H-7a, d, $J_{7a,b}$ = 12.9); 4.2 (H-7b, d, $J_{7b,a}$ = 12.9); 4.0 (H-4, d, $J_{4,3}$ = 8.9); 3.8 (H-1 and H-2, m); 3.6 (H-3, t, $J_{3,4}$ = 8.9); 1.8 (-CH_3/Ac, s).

^{13}C NMR (100MHz, D_2O): δ (ppm) 181.8 (>C=O/Ac); 136.1 (C-5); 127.9 (C-2'); 125.9 (C-6'); 124.3 (C-7'); 120.1 (C-6); 118.7 (C-8'); 116.3 (C-5'); 72.1 (C-3); 70.7 (C-4); 67.0 (C-2); 55.2 (C-1); 40.0 (C-7); 23.3 (-CH_3/Ac).

```
P53341|MAL12_YEAST              MTIS-DHPETEPKWWKEATIYQIYPASFKDSNNDGWGDLKGITSKLQYIK 49
3AJ7_A|PDBID|CHAIN|SEQUENCE     MTISSAHPETEPKWWKEATFYQIYPASFKDSNDDGWGDMKGIASKLEYIK 50
                                ****  *************:************:*****:***:***:***

P53341|MAL12_YEAST              DLGVDAIWVCPFYDSPQQDMGYDISNYEKVWPTYGTNEDCFELIDKTHKL 99
3AJ7_A|PDBID|CHAIN|SEQUENCE     ELGADAIWISPFYDSPQDDMGYDIANYEKVWPTYGTNEDCFALIEKTHKL 100
                                :**.****:.*******:******:**************** **:*****

P53341|MAL12_YEAST              GMKFITDLVINHCSTEHEWFKESRSSKTNPKRDWFFWRPPKGYDAEGKPI 149
3AJ7_A|PDBID|CHAIN|SEQUENCE     GMKFITDLVINHCSSEHEWFKESRSSKTNPKRDWFFWRPPKGYDAEGKPI 150
                                **************:***********************************

P53341|MAL12_YEAST              PPNNWKSFFGGSAWTFDETTNEFYLRLFASRQVDLNWENEDCRRAIFESA 199
3AJ7_A|PDBID|CHAIN|SEQUENCE     PPNNWKSYFGGSAWTFDEKTQEFYLRLFCSTQPDLNWENEDCRKAIYESA 200
                                *******:**********.*:*******.* * **********:**:***

P53341|MAL12_YEAST              VGFWLDHGVDGFRIDTAGLYSKRPGLPDSPIFDKTSKLQHPNWGSHNGPR 249
3AJ7_A|PDBID|CHAIN|SEQUENCE     VGYWLDHGVDGFRIDVGSLYSKVVGLPDAPVVDKNSTWQSSDPYTLNGPR 250
                                **:************...****  ****:*:.**.*. * .:  : ****

P53341|MAL12_YEAST              IHEYHQELHRFMKNRVKDGREIMTVGEVAHGSDN--ALYTSAARYEVSEV 297
3AJ7_A|PDBID|CHAIN|SEQUENCE     IHEFHQEMNQFIRNRVKDGREIMTVGEMQHASDETKRLYTSASRHELSEL 300
                                ***:***:::*::**************: *.**:   *****:*:*:**:

P53341|MAL12_YEAST              FSFTHVEVGTSPFFRYNIVPFTLKQWKEAIASNFLFINGTDSWATTYIEN 347
3AJ7_A|PDBID|CHAIN|SEQUENCE     FNFSHTDVGTSPLFRYNLVPFELKDWKIALAELFRYINGTDCWSTIYLEN 350
                                *.*:*.:*****:****:*** **:** *:*. * :*****.*:* *:**

P53341|MAL12_YEAST              HDQARSITRFADDSPKYRKISGKLLTLLECSLTGTLYVYQGQEIGQINFK 397
3AJ7_A|PDBID|CHAIN|SEQUENCE     HDQPRSITRFGDDSPKNRVISGKLLSVLLSALTGTLYVYQGQELGQINFK 400
                                ***.******.***** * ******::* .:************:******

P53341|MAL12_YEAST              EWPIEKYEDVDVKNNYEIIKKSFGKNSKEMKDFFKGIALLSRDHSRTPMP 447
3AJ7_A|PDBID|CHAIN|SEQUENCE     NWPVEKYEDVEIRNNYNAIKEEHGENSEEMKKFLEAIALISRDHARTPMQ 450
                                :**:******:::***: **:..*:**:***.*::.***:****:****

P53341|MAL12_YEAST              WTKDKPNAGFTGPDVKPWFLLNESFEQGINVEQESRDDDSVLNFWKRALQ 497
3AJ7_A|PDBID|CHAIN|SEQUENCE     WSREEPNAGFSGPSAKPWFYLNDSFREGINVEDEIKDPNSVLNFWKEALK 500
                                *::::*****:**..**** **:**.:*****:* :* :*******.**:

P53341|MAL12_YEAST              ARKKYKELMIYGYDFQFIDLDSDQIFSFTKEYEDKTLFAALNFSGEEIEF 547
3AJ7_A|PDBID|CHAIN|SEQUENCE     FRKAHKDITVYGYDFEFIDLDNKKLFSFTKKYNNKTLFAALNFSSDATDF 550
                                 ** :*:: :*****:*****..::*****:*::**********.:  :*

P53341|MAL12_YEAST              SLPREGASLSFILGNY--DDTDVSSRVLKPWEGRIYLVK 584
3AJ7_A|PDBID|CHAIN|SEQUENCE     KIPNDDSSFKLEFGNYPKKEVDASSRTLKPWEGRIYISE 589
                                .:*.:.:*:.: :***  .:.*.***.*********: :
```

Sequence alignment displaying the homology between MAL12 (P53341) and oligo-1,6-glucosidase (PDB code 3AJ7) both from *Saccharomyces cerevisiae* (PDB code 3AJ7).

Compound 1: (+)-(1S,2S,3S,4R)-1-aminociclohex-5-en-2,3,4-triol

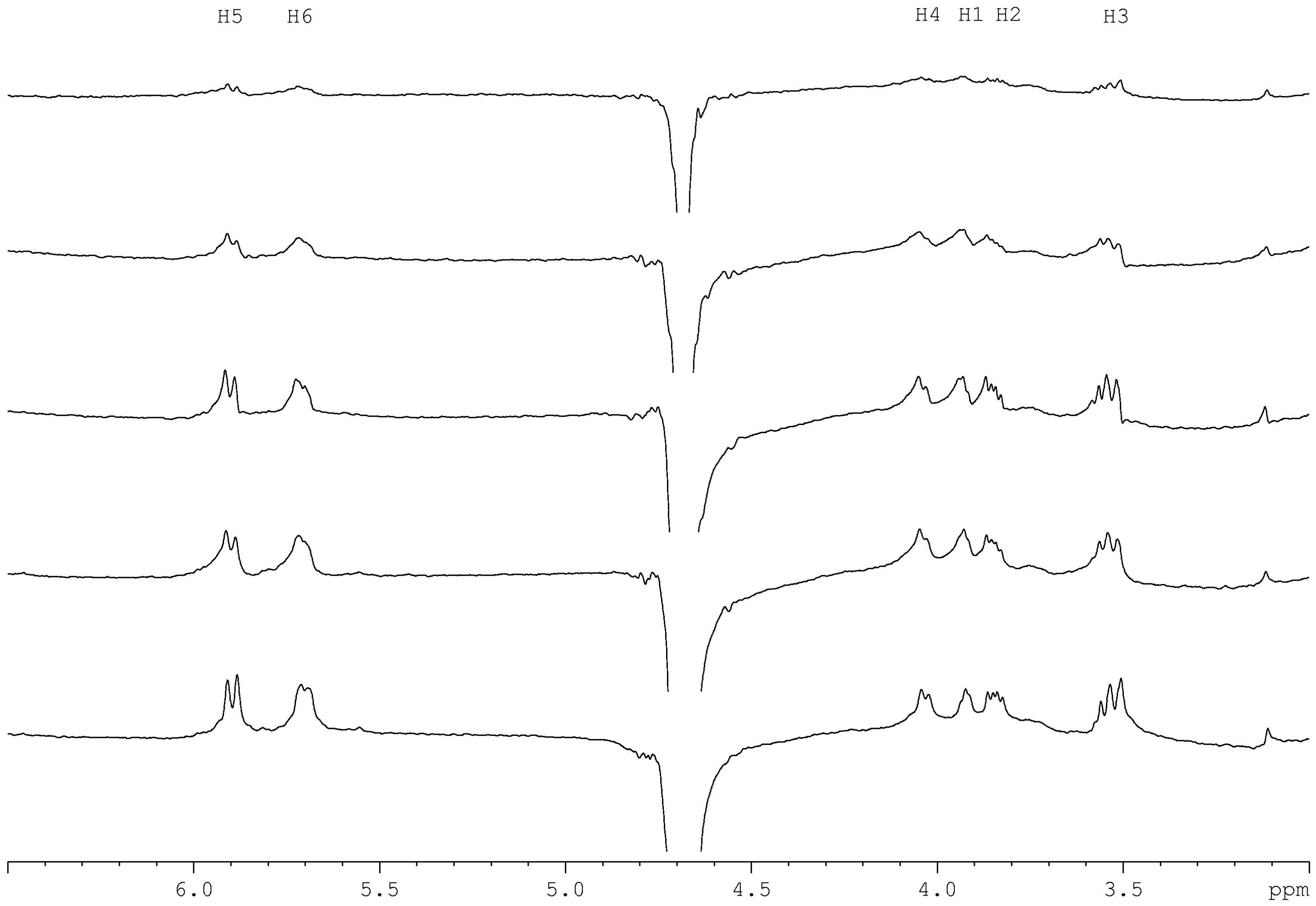

Figure S1: STD-NMR spectra of compound **1** in complex with α-1,4-glucosidase (200:1 molar excess) at different saturation times (500 ms, 1s, 2s, 3s and 5 s from the top to the bottom)

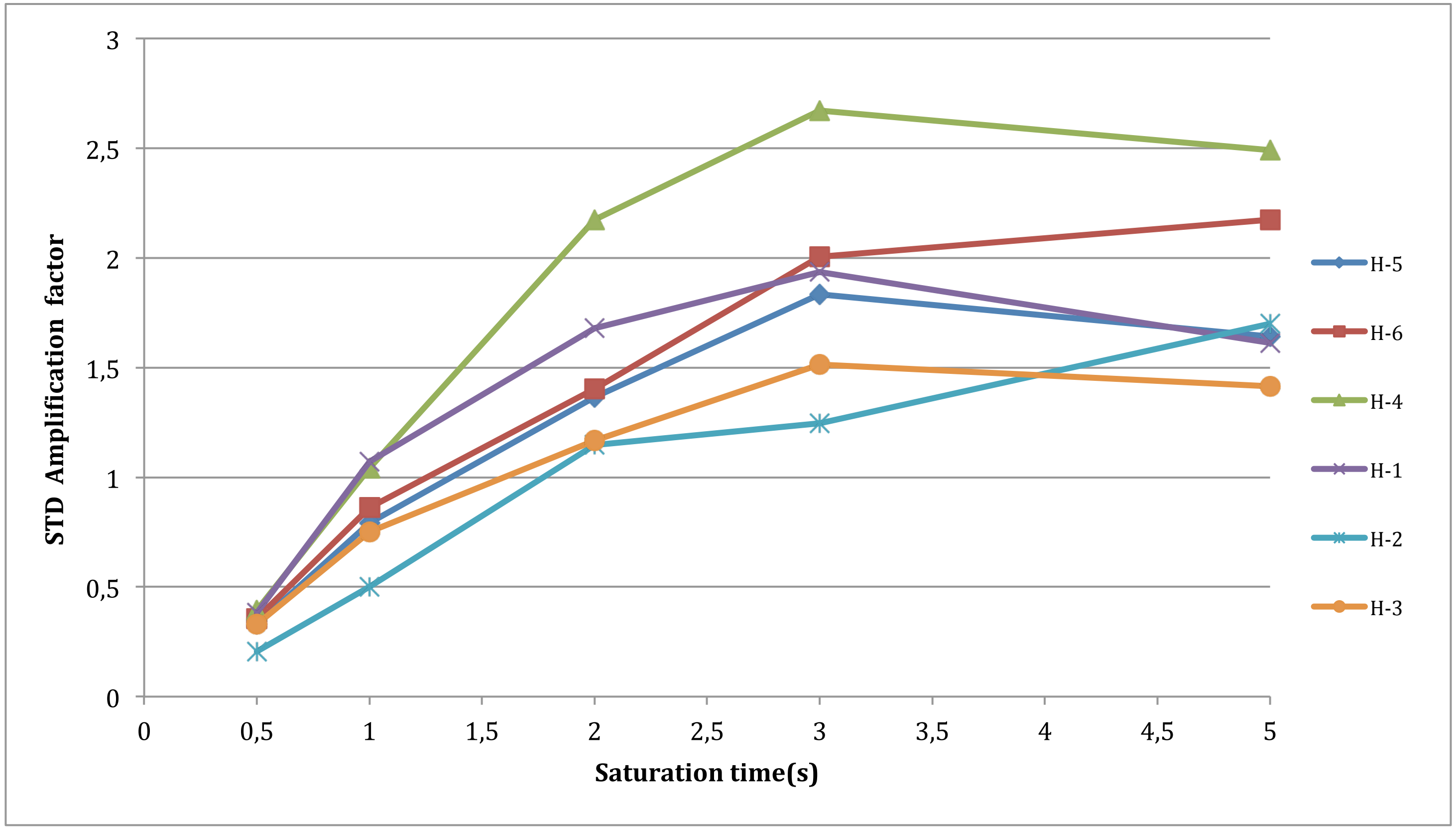

Figure S2: Plot of the STD amplification factor for each proton resonance as a function of the applied saturation time for compound **1** in complex with α-1,4-glucosidase.

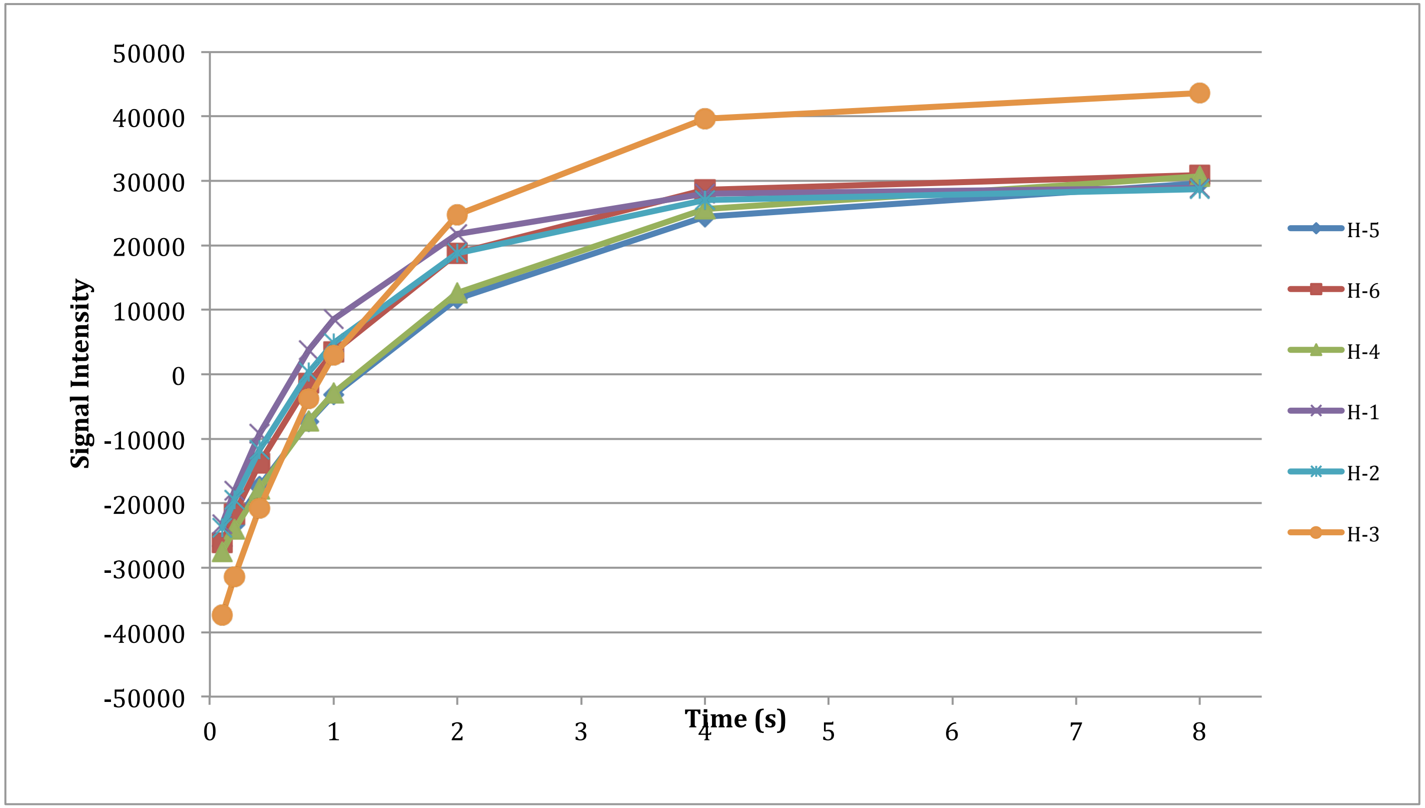

Figure S3: Plot of the signal intensity measured for each proton resonance of compound **1** as a function of the recovery time used in an inversion-recovery pulse sequence. Compound **1** was in a 200:1 molar excess with respect to α-1,4-glucosidase.

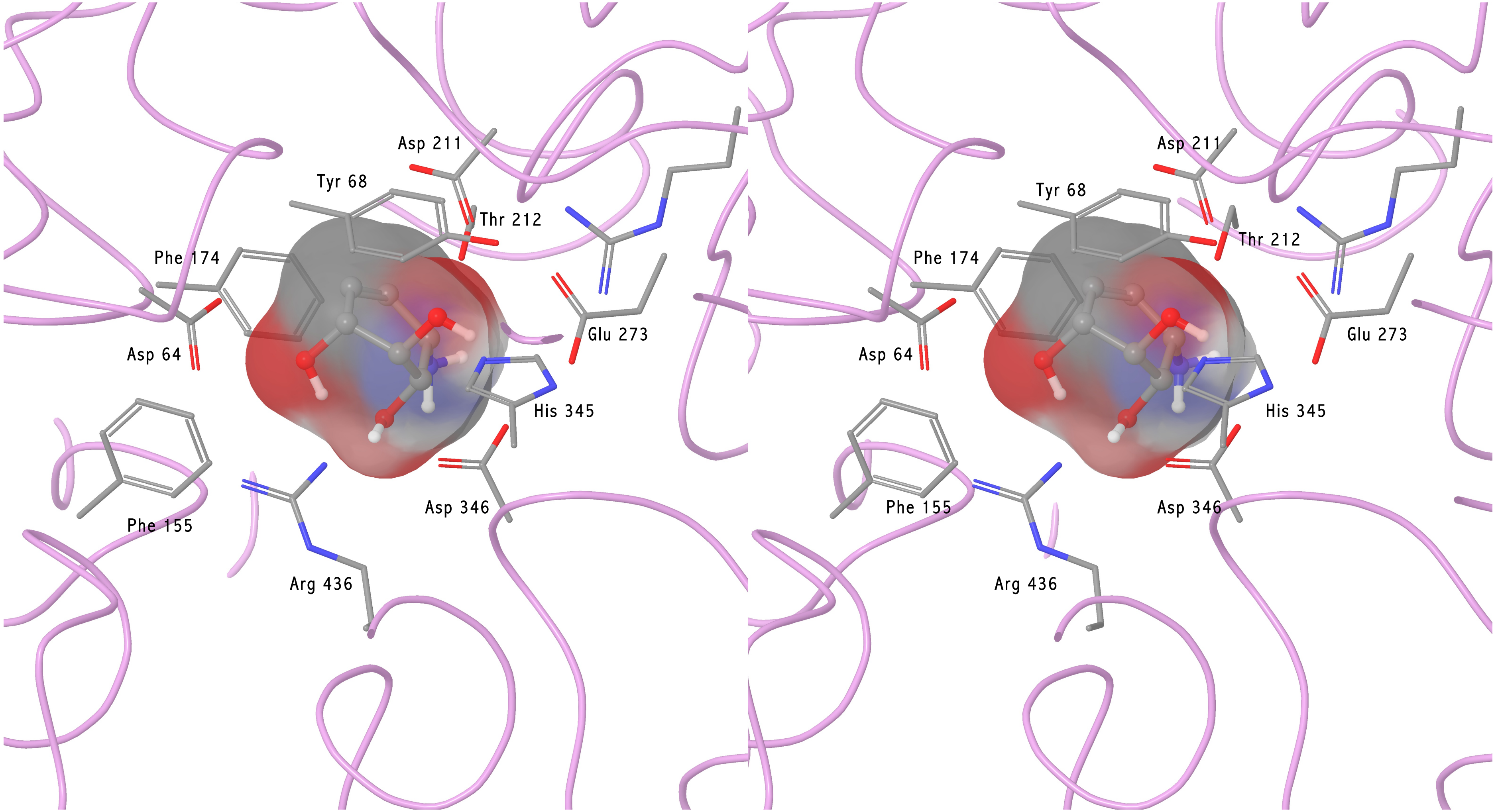

Figure S4: Stereo view of the modelled α-1,4-glucosidase active site in complex with the docked structure of compound **1**. The ligand is surrounded by a Van der Waals surface and only those aminoacid residues within a distance of 4 Å of the inhibitor are shown.

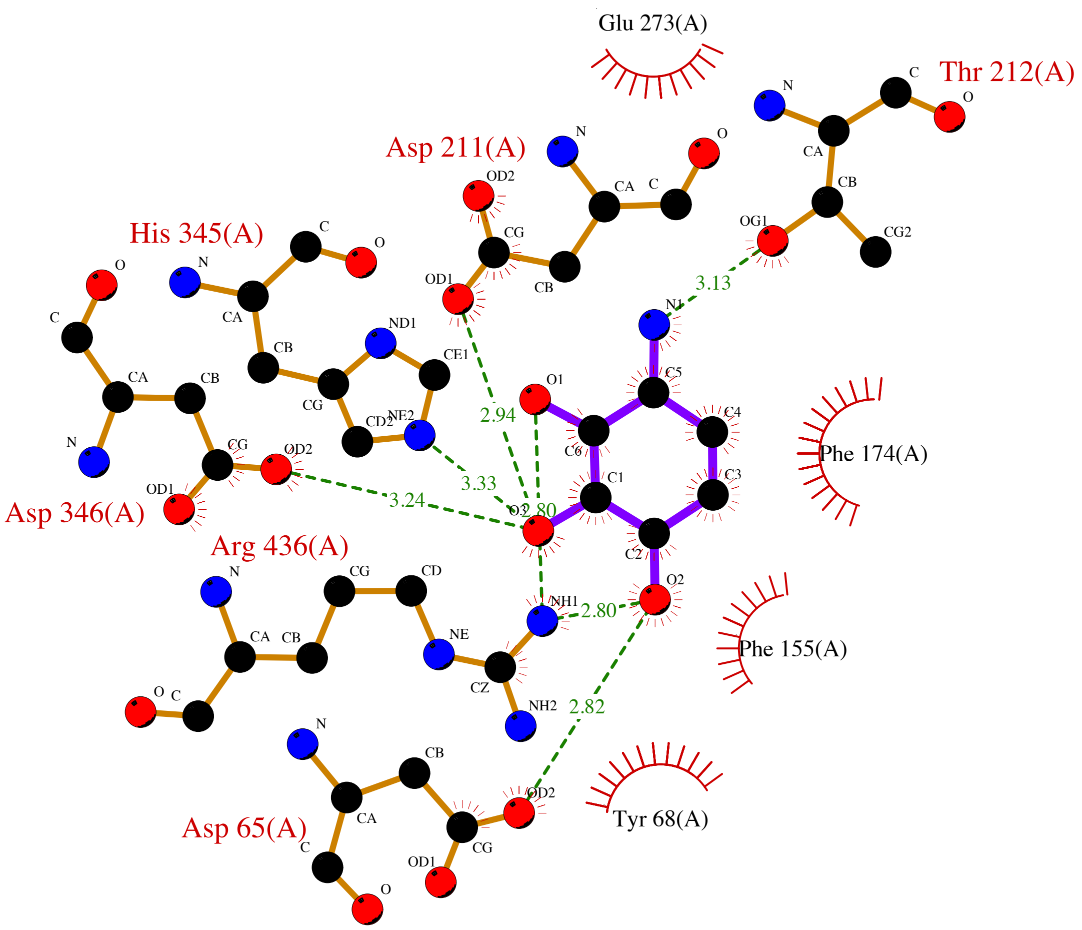

Figure S5: Ligplot cartoon illustrating the interactions between the docked structure of compound **1** and the modelled α-1,4-glucosidase active site.

Compound 2: (+)-(1S,2S,3S,4R)-1-[(4hidroxibencil)amino]ciclohex-5-en-2,3,4-triol

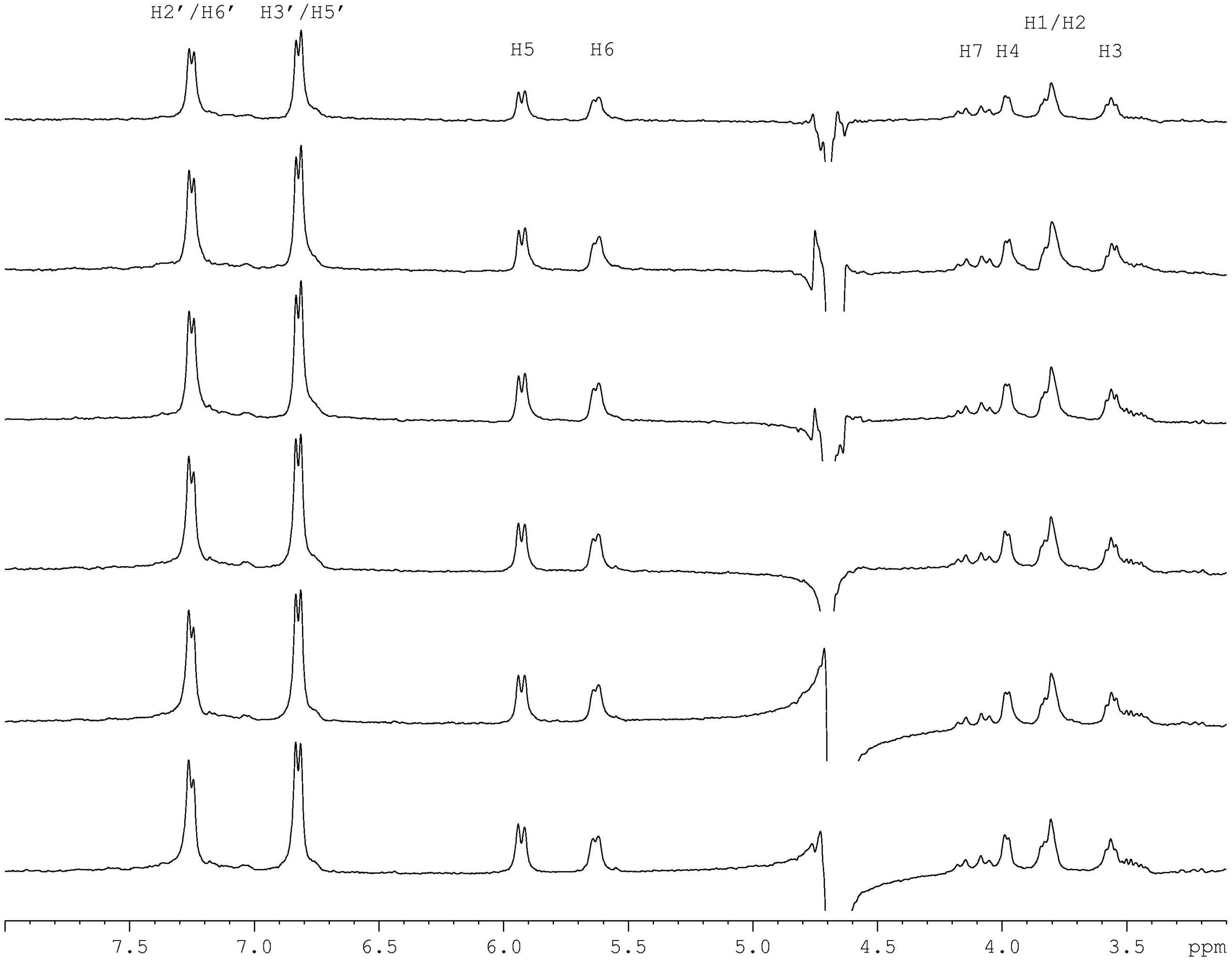

Figure S6: STD-NMR spectra of compound **2** in complex with α-1,4-glucosidase (200:1 molar excess) at different saturation times (250 ms, 500 ms, 1s, 2s, 3s and 5 s from the top to the bottom).

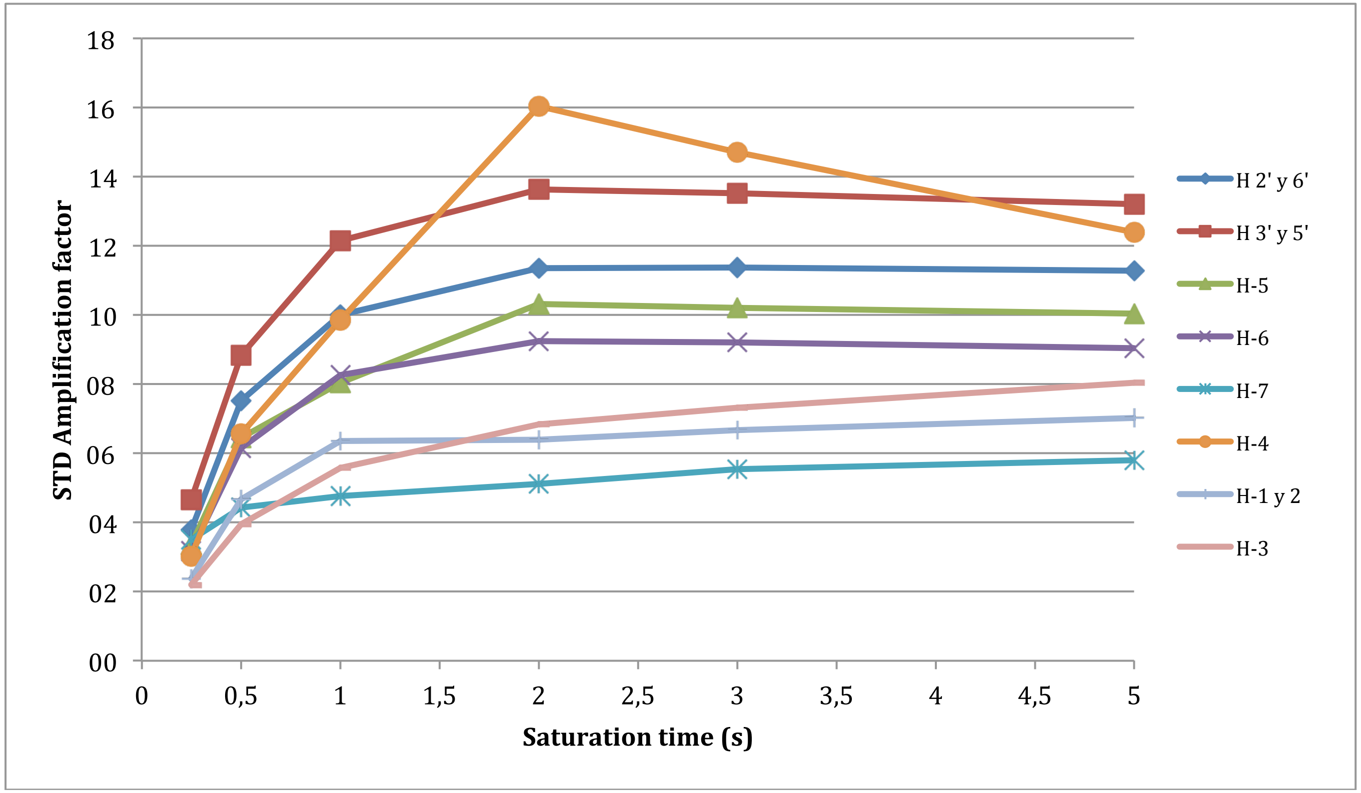

Figure S7: Plot of the STD amplification factor for each proton resonance as a function of the applied saturation time for compound **2** in complex with α-1,4-glucosidase.

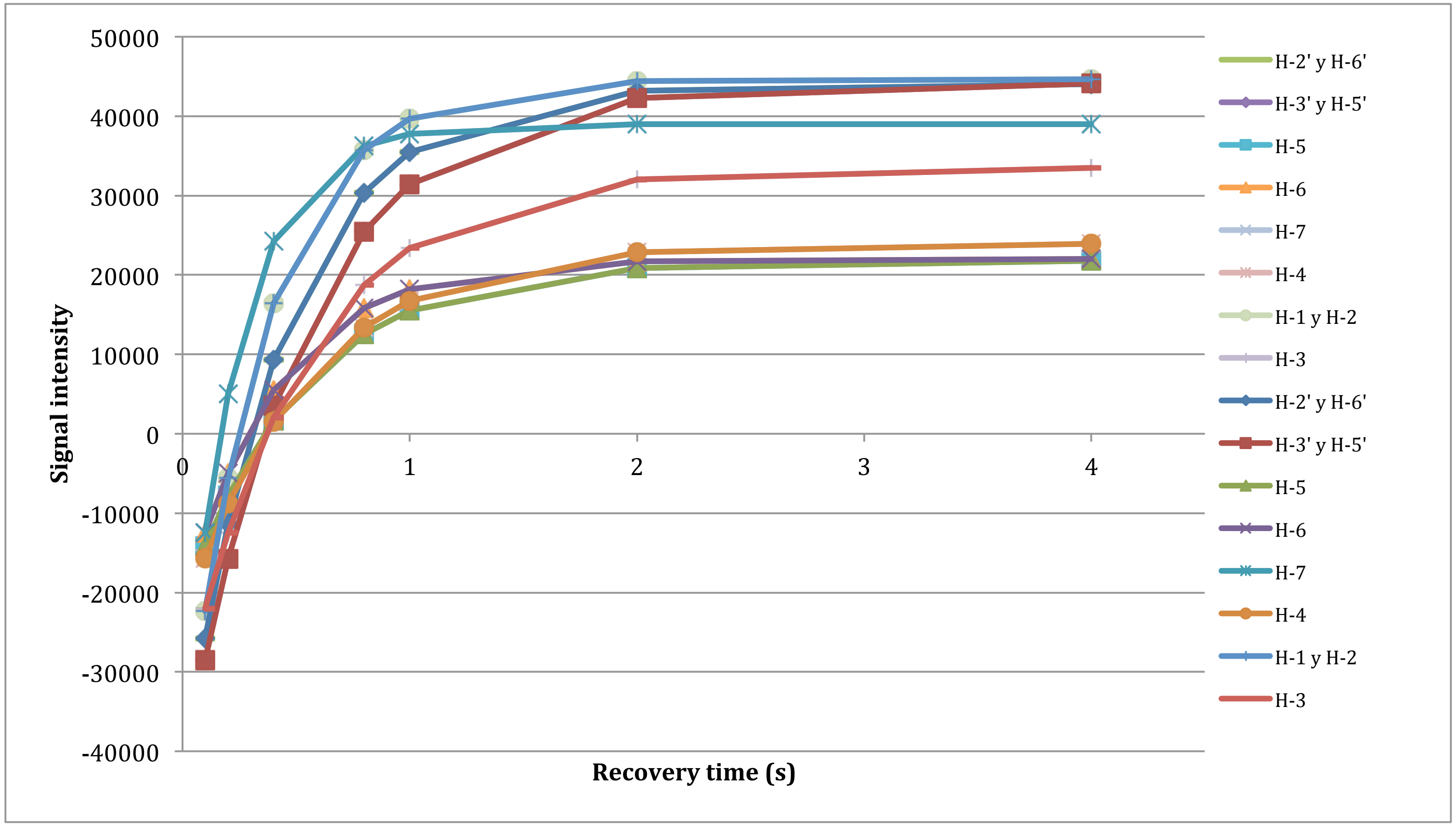

Figure S8: Plot of the signal intensity measured for each proton resonance of compound **2** as a function of the recovery time used in an inversion-recovery pulse sequence. Compound **2** was in a 200:1 molar excess with respect to α-1,4-glucosidase.

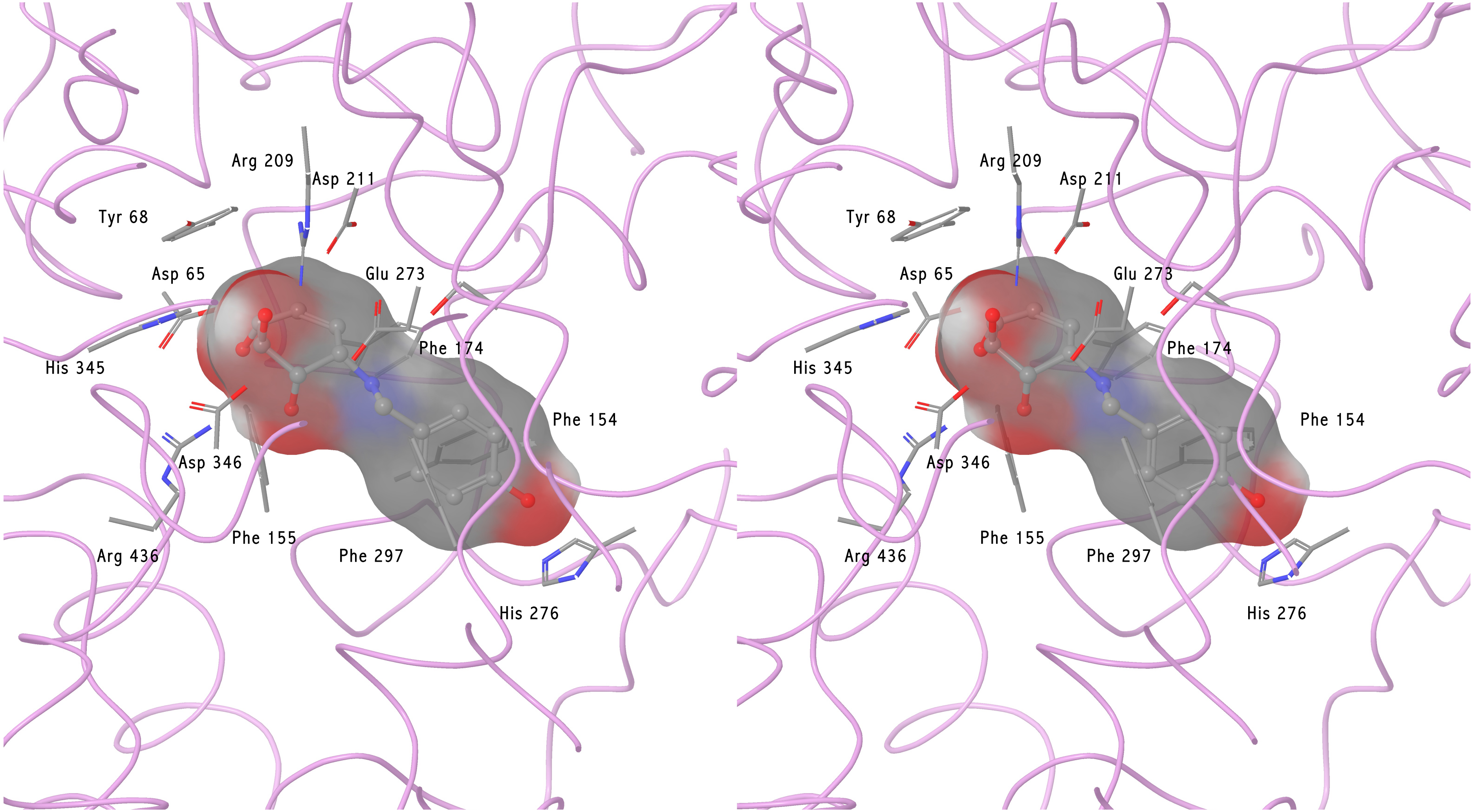

Figure S9: Stereo view of the α-1,4-glucosidase active site in complex with the docked structure of compound **2**. The ligand is surrounded by a Van der Waals surface and only those aminoacid residues within a distance of 4 Å of the inhibitor are shown.

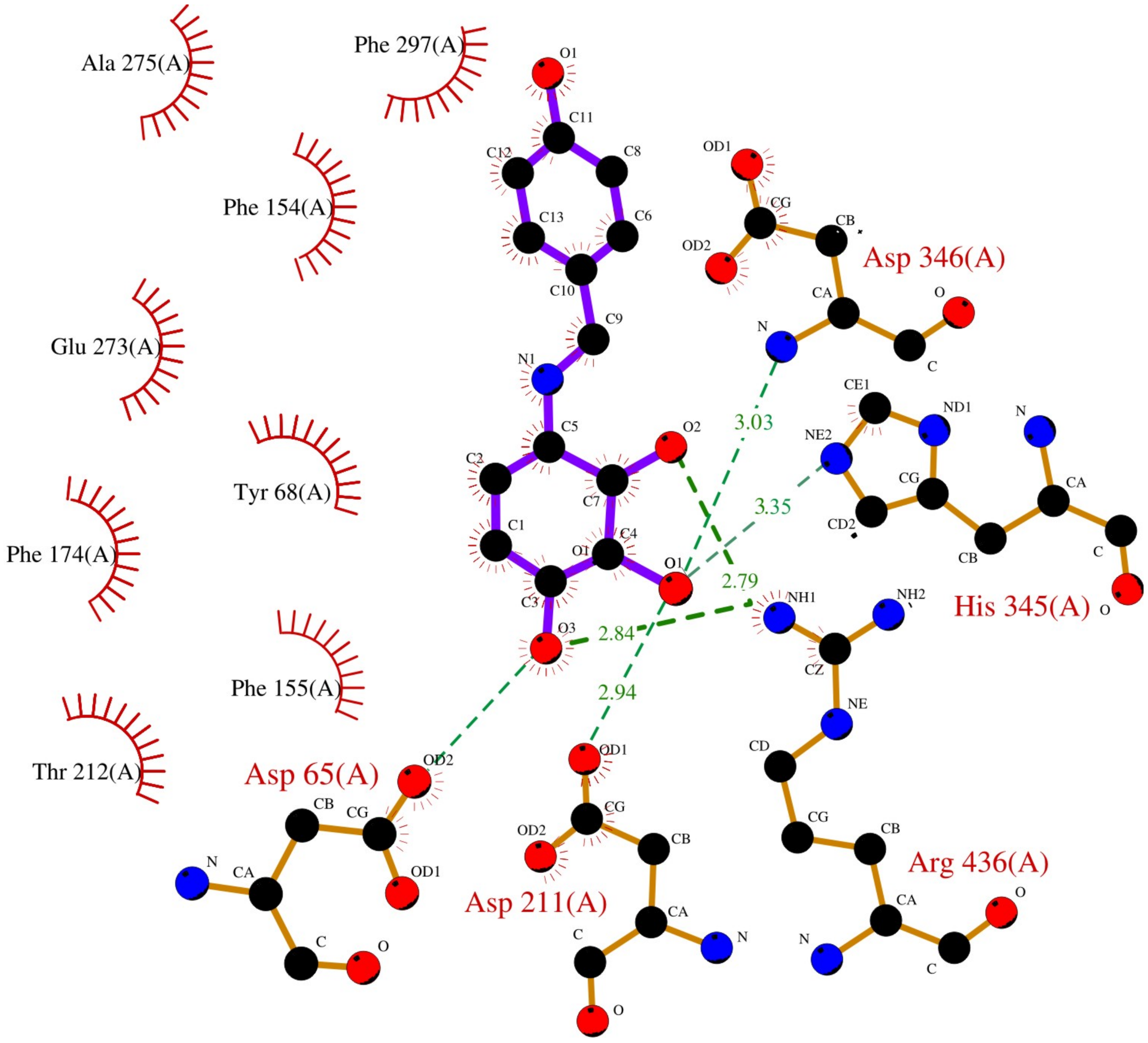

Figure S10: Ligplot cartoon illustrating the interactions between the docked structure of compound **2** and the modelled α-1,4-glucosidase active site.

Compound 3: (+)-(1S,2S,3S,4R)-1-[(4-piridin-4-il-bencil)amino]ciclohex-5-en-2,3,4-triol

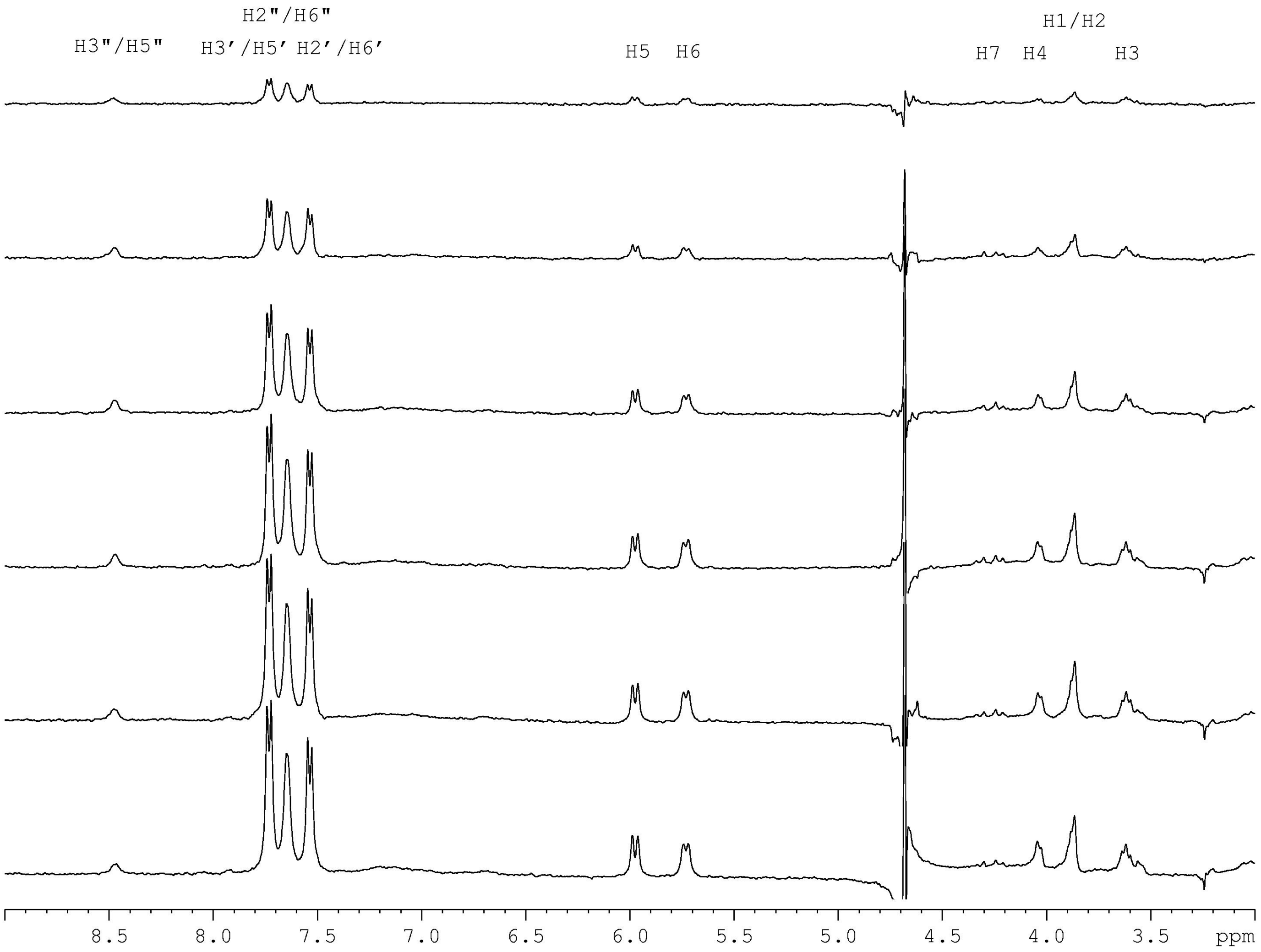

Figure S11: STD-NMR spectra of compound **3** in complex with α-1,4-glucosidase (200:1 molar excess) at different saturation times (250 ms, 500 ms, 1s, 2s, 3s and 5 s from the top to the bottom).

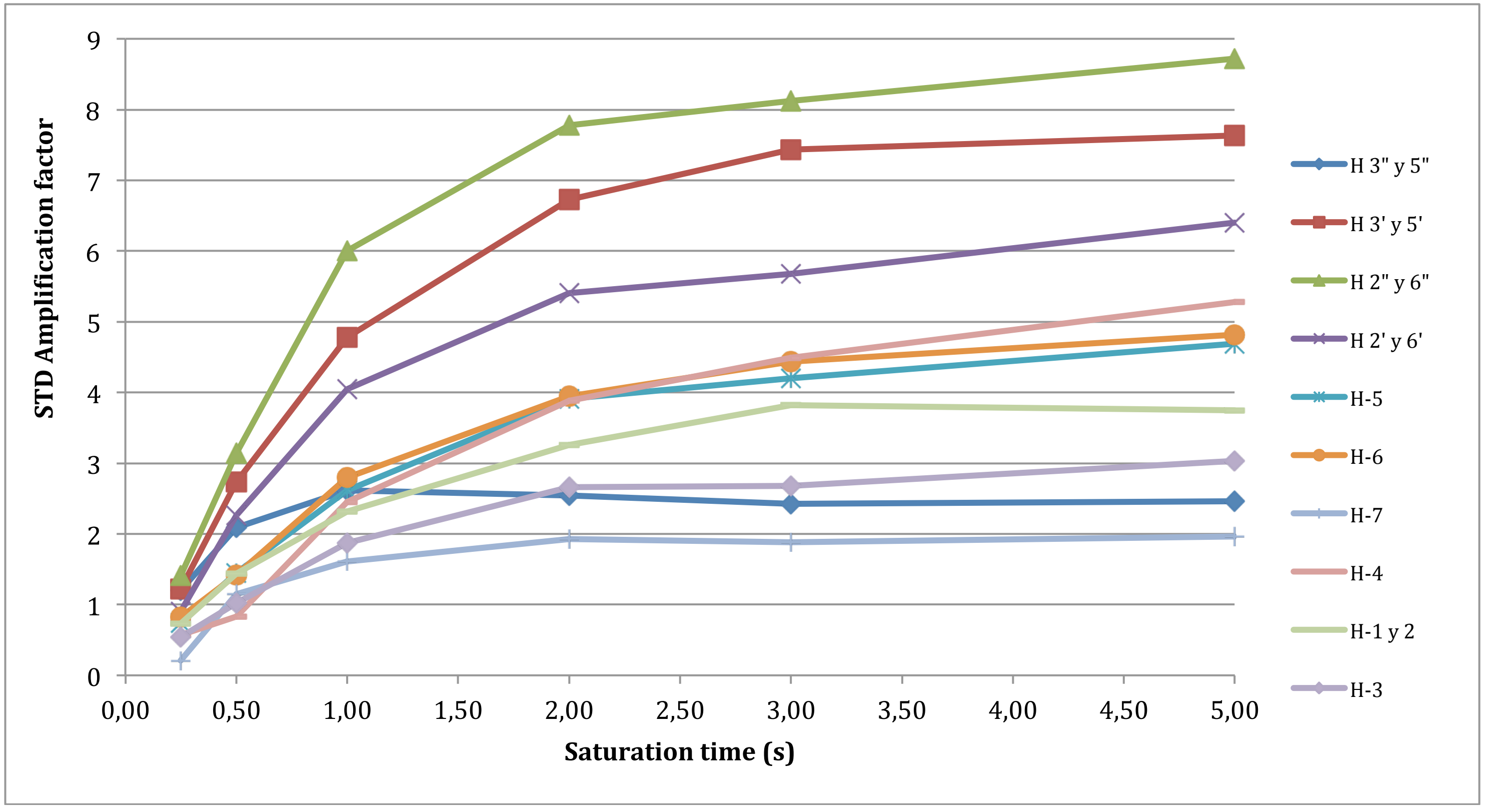

Figure S12: Plot of the STD amplification factor for each proton resonance as a function of the applied saturation time for compound **3** in complex with α-1,4-glucosidase.

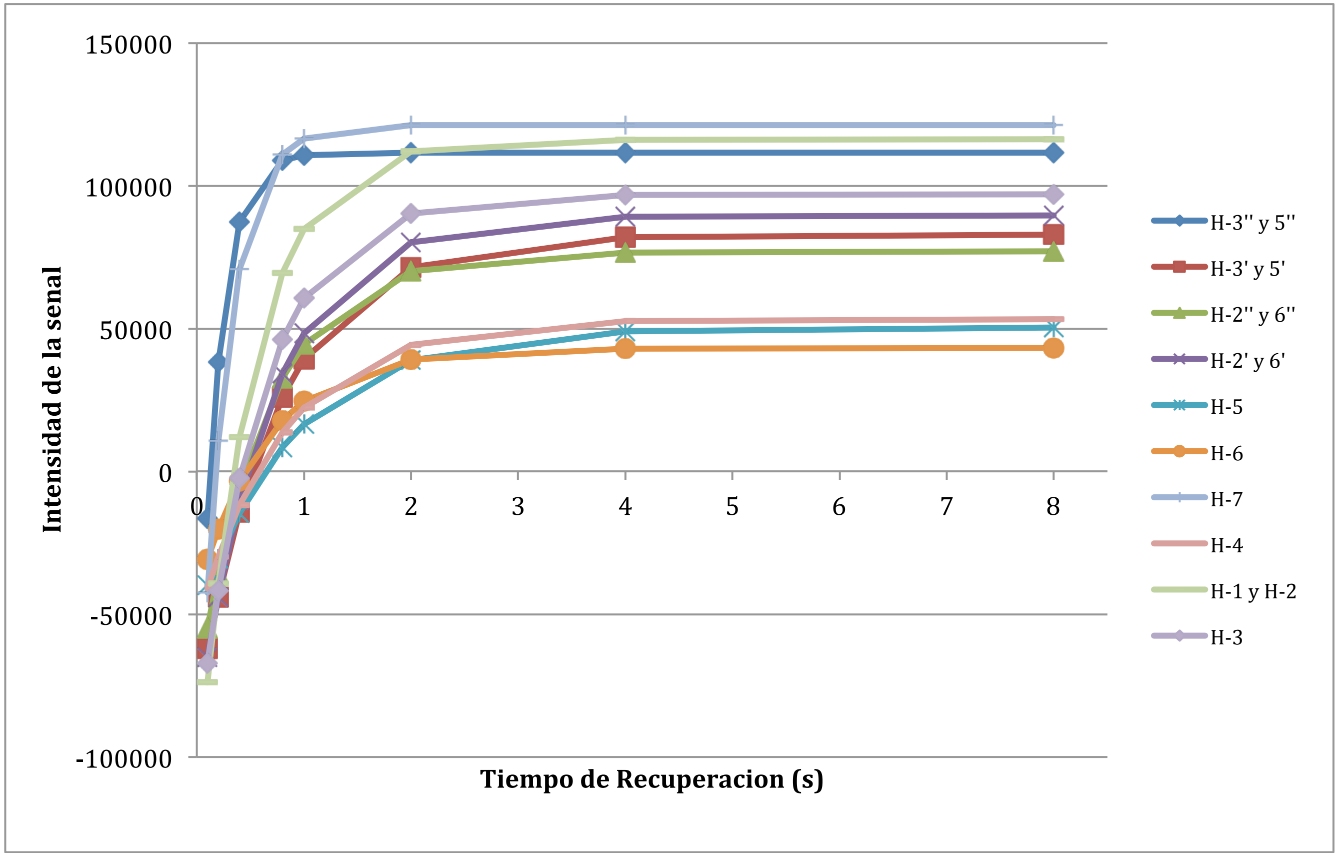

Figure S13: Plot of the signal intensity measured for each proton resonance of compound **3** as a function of the recovery time used in an inversion-recovery pulse sequence. Compound **3** was in a 200:1 molar excess with respect to α-1,4-glucosidase.

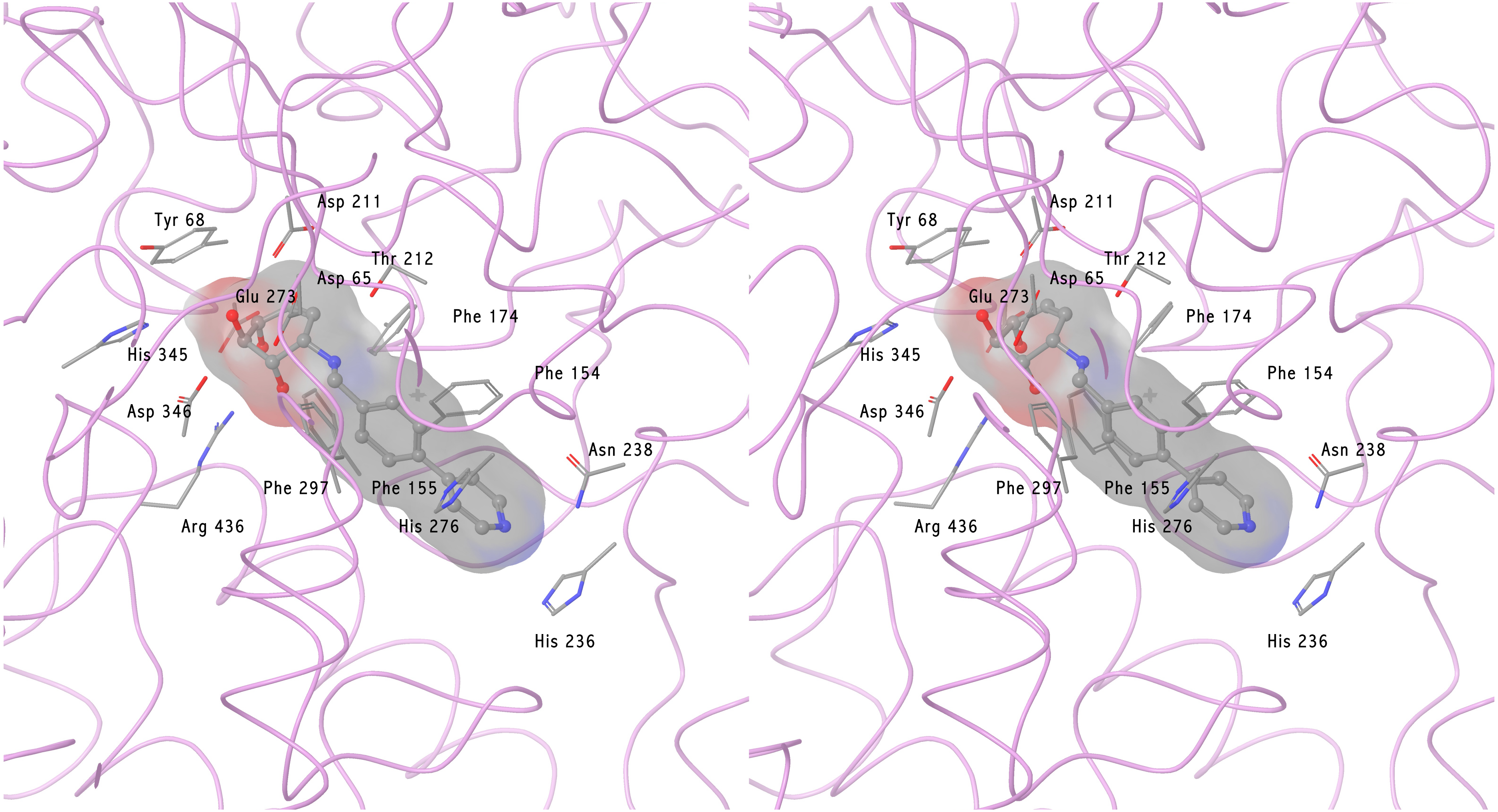

Figure S14: Stereo view of the α-1,4-glucosidase active site in complex with the docked structure of compound **3**. The ligand is surrounded by a Van der Waals surface and only those aminoacid residues within a distance of 4 Å of the inhibitor are shown.

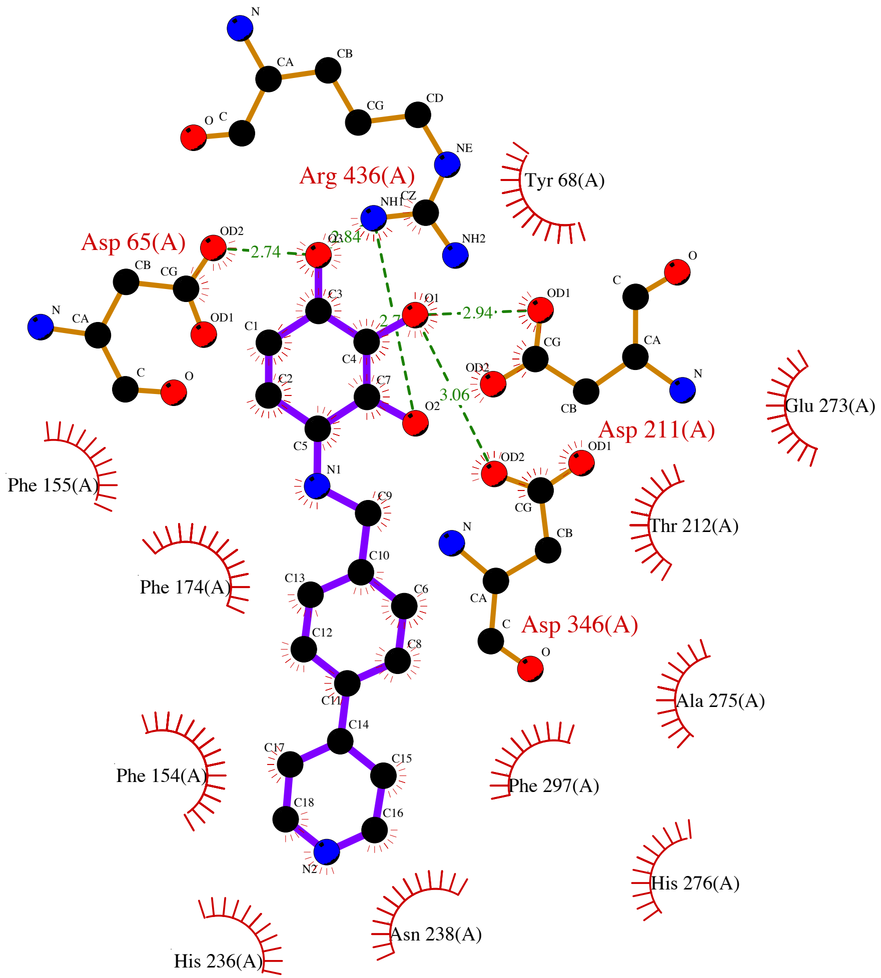

Figure S15: Ligplot cartoon illustrating the interactions between the docked structure of compound **3** and the modelled α-1,4-glucosidase active site.

Compound 4: (+)-(1S,2S,3S,4R)-1-{[(1-acetil-1H-indol-3-il)]methyl}amino}ciclohex-5-en-2,3,4-triol

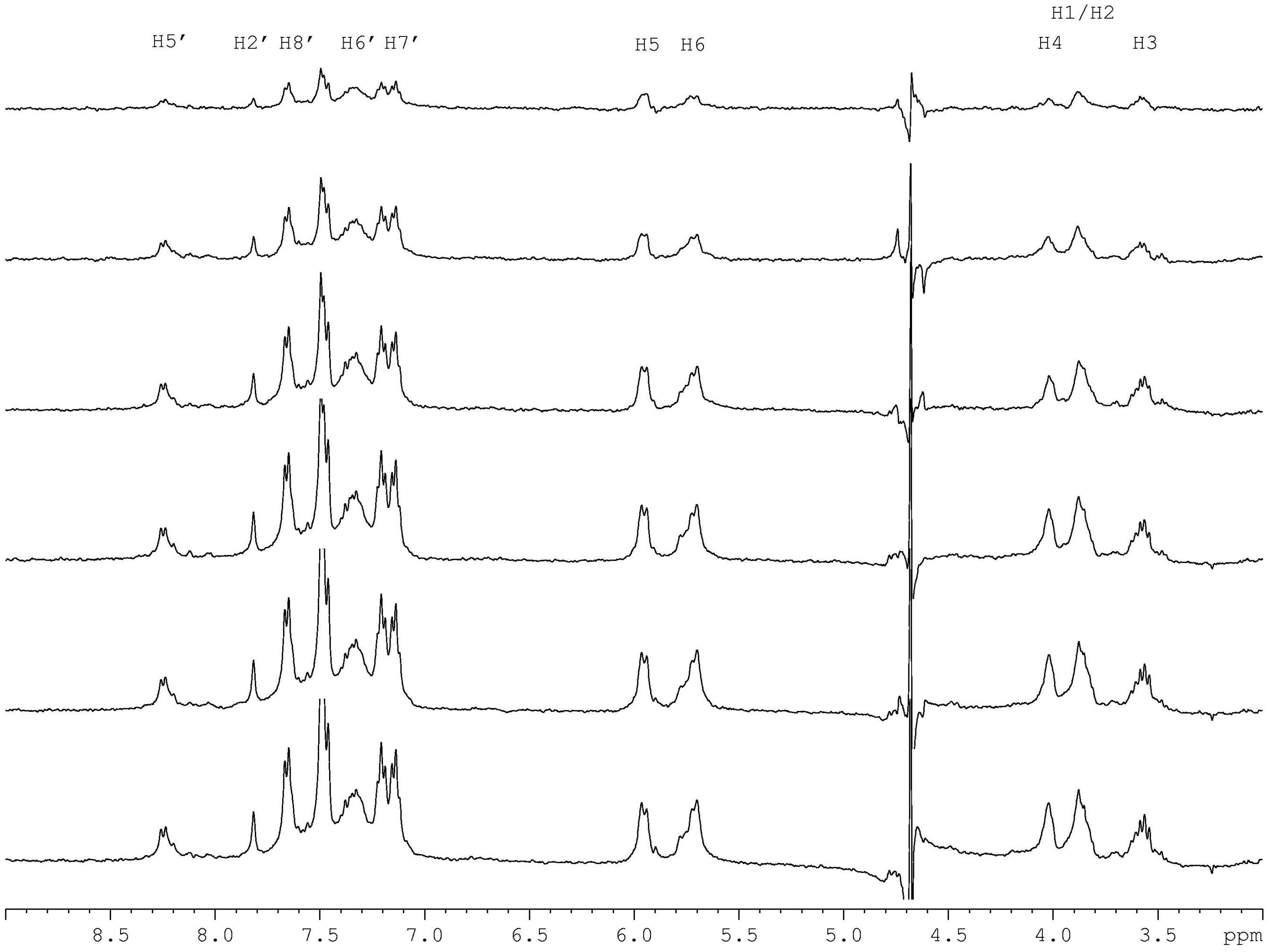

Figure S16: STD-NMR spectra of compound **4** in complex with α-1,4-glucosidase (200:1 molar excess) at different saturation times (250 ms, 500 ms, 1s, 2s, 3s and 5 s from the top to the bottom).

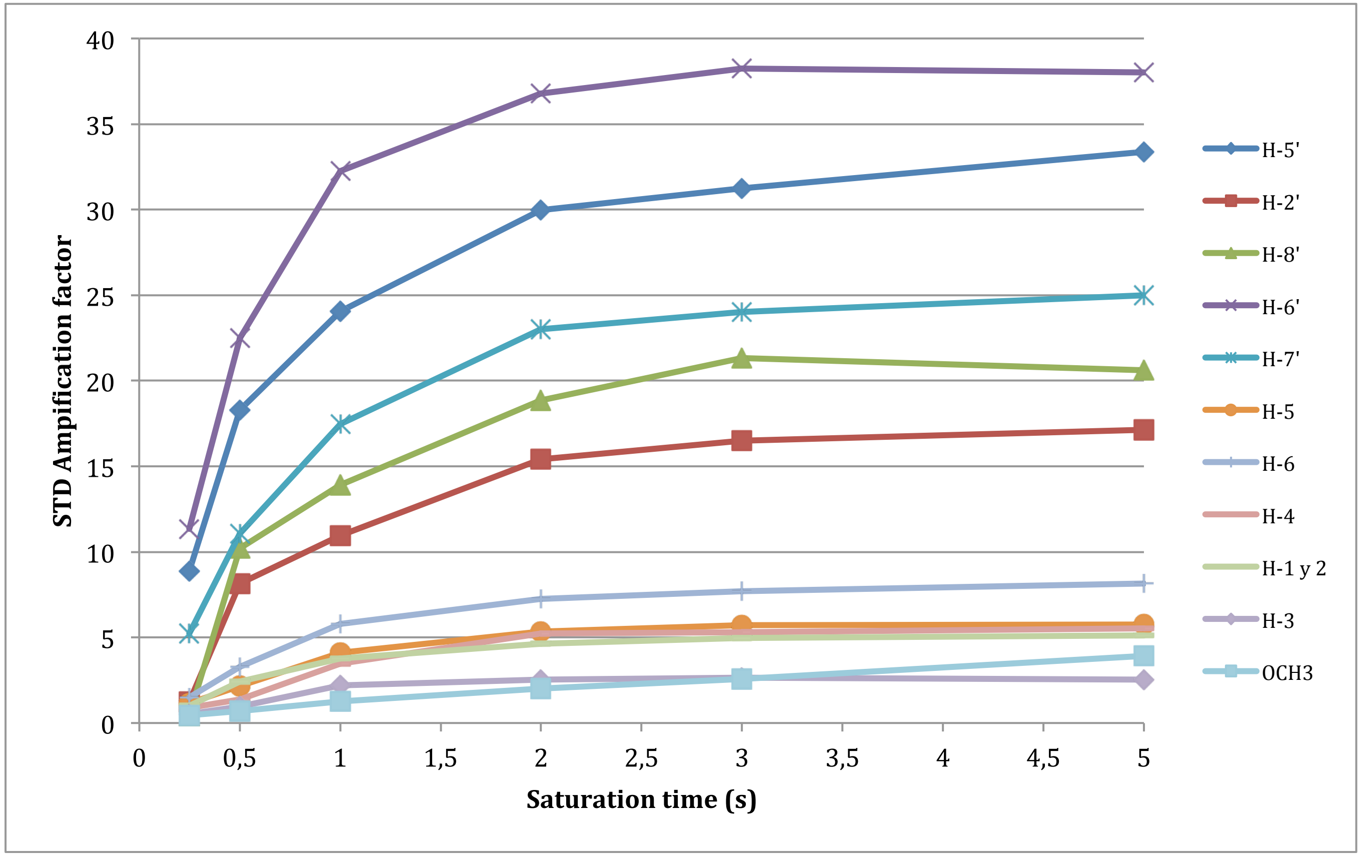

Figure S17: Plot of the STD amplification factor for each proton resonance as a function of the applied saturation time for compound **4** in complex with α-1,4-glucosidase.

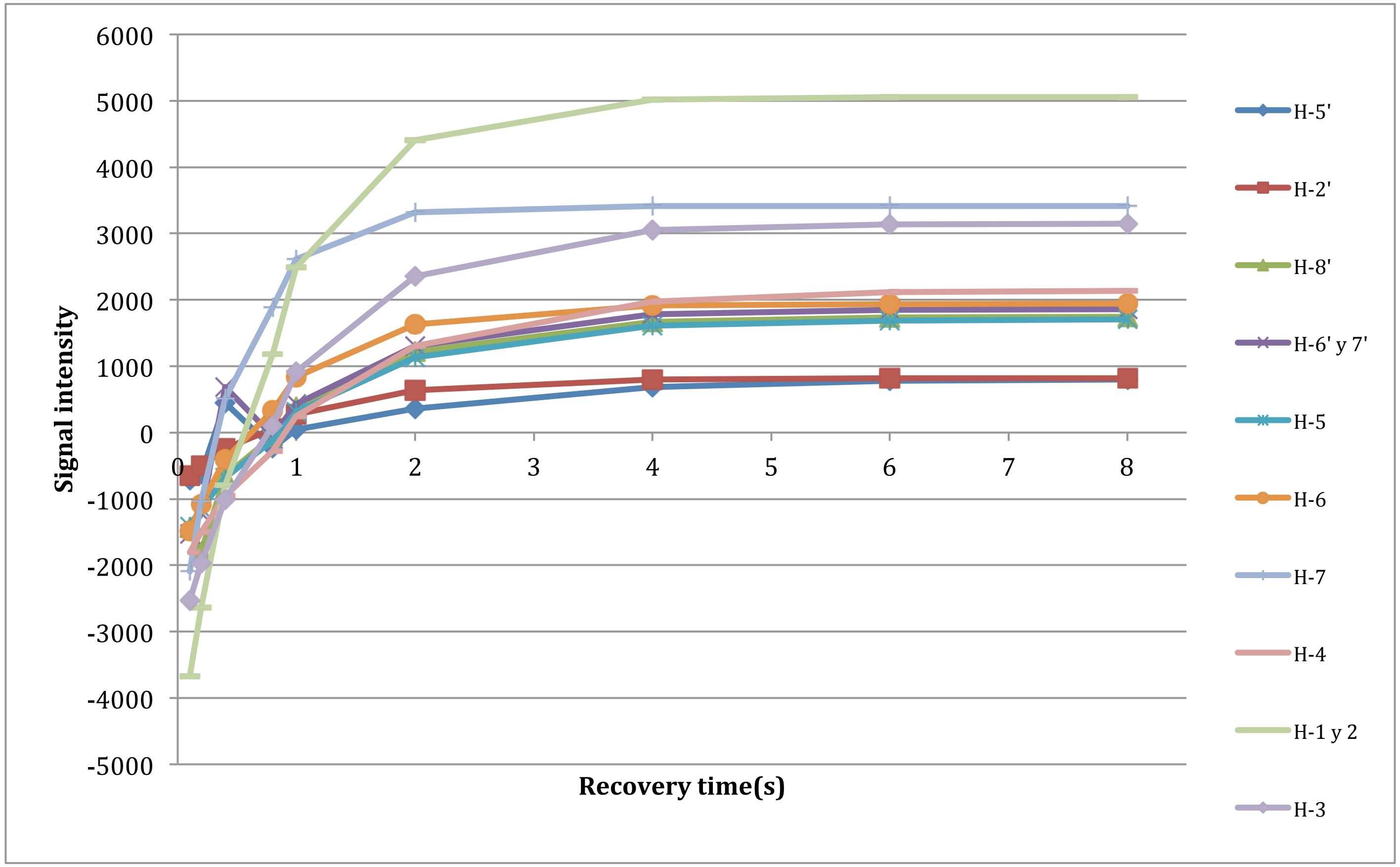

Figure S18: Plot of the signal intensity measured for each proton resonance of compound **4** as a function of the recovery time used in an inversion-recovery pulse sequence. Compound **4** was in a 200:1 molar excess with respect to α-1,4-glucosidase.

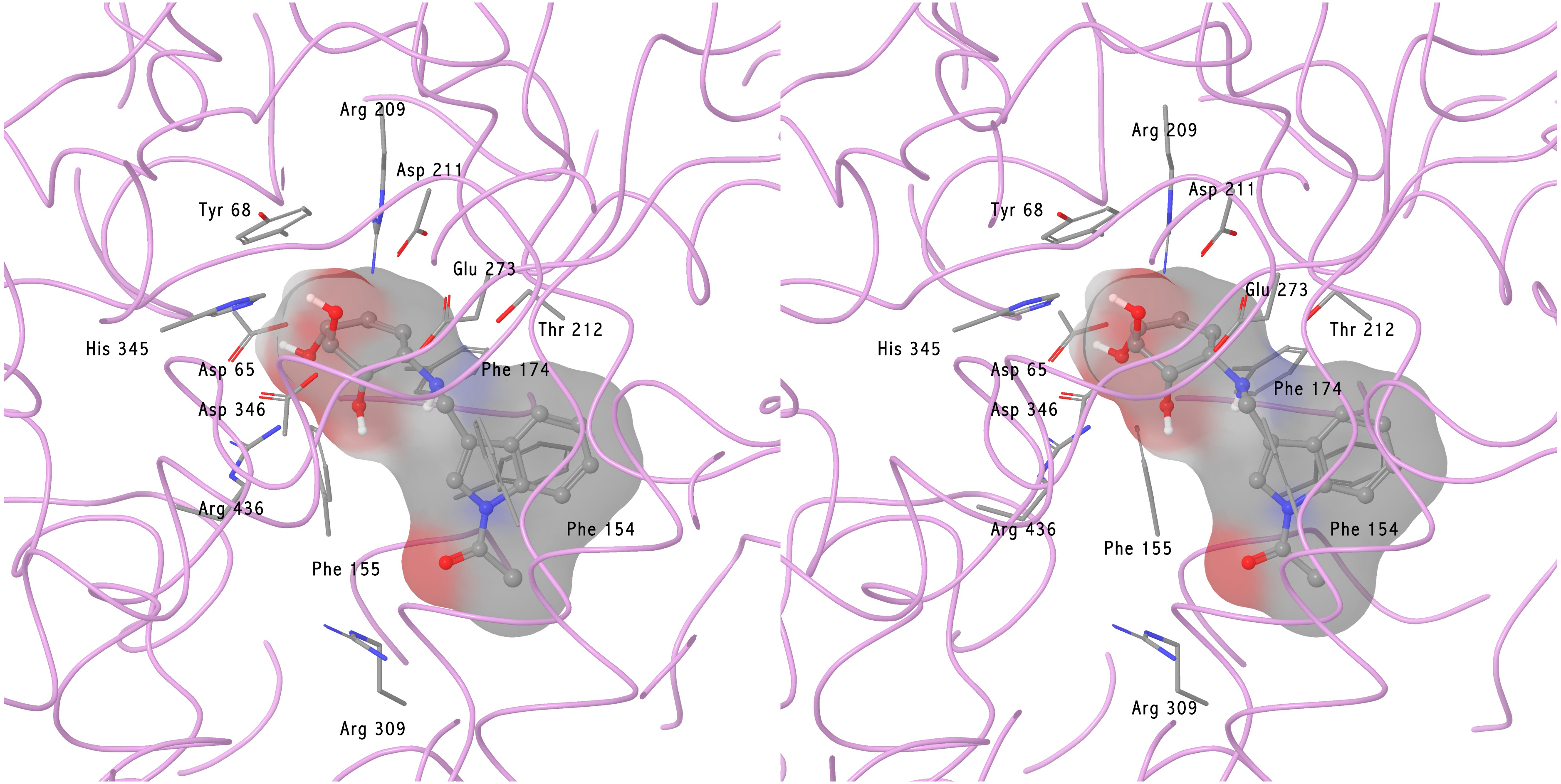

Figure S19: Stereo view of the α-1,4-glucosidase active site in complex with the docked structure of compound **4**. The ligand is surrounded by a Van der Waals surface and only those aminoacid residues within a distance of 4 Å of the inhibitor are shown.

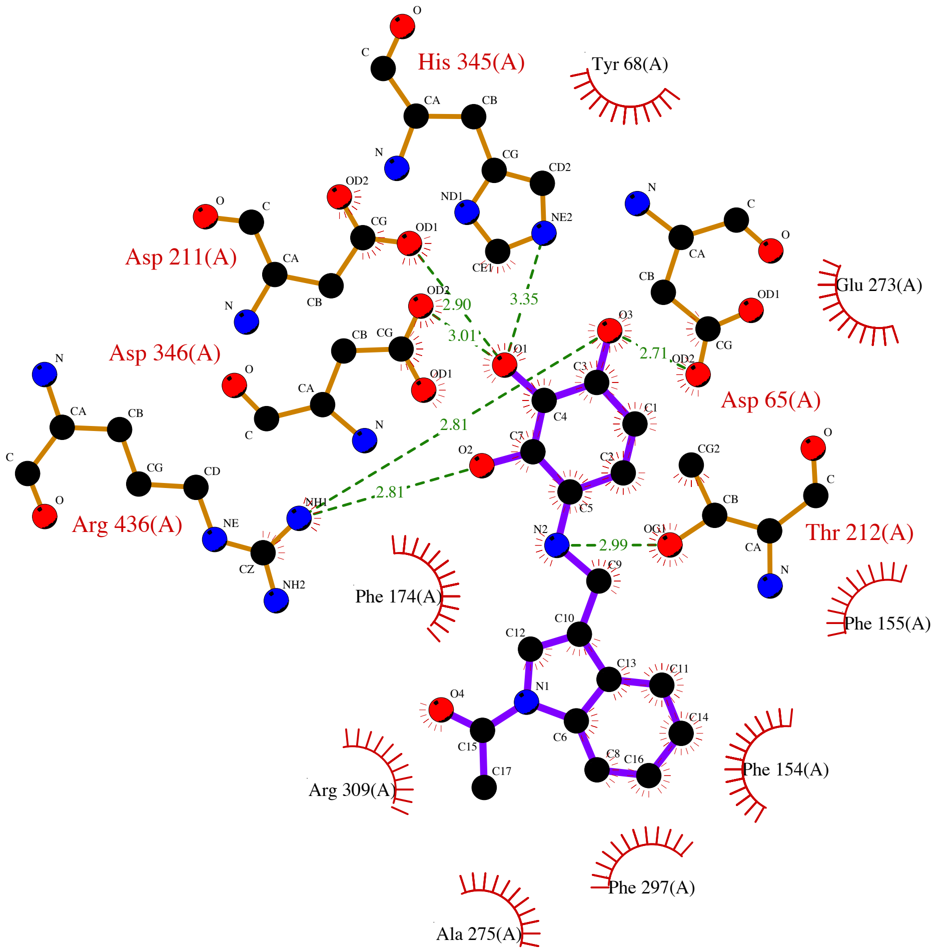

Figure S20: Ligplot cartoon illustrating the interactions between the docked structure of compound **4** and the modelled α-1,4-glucosidase active site.

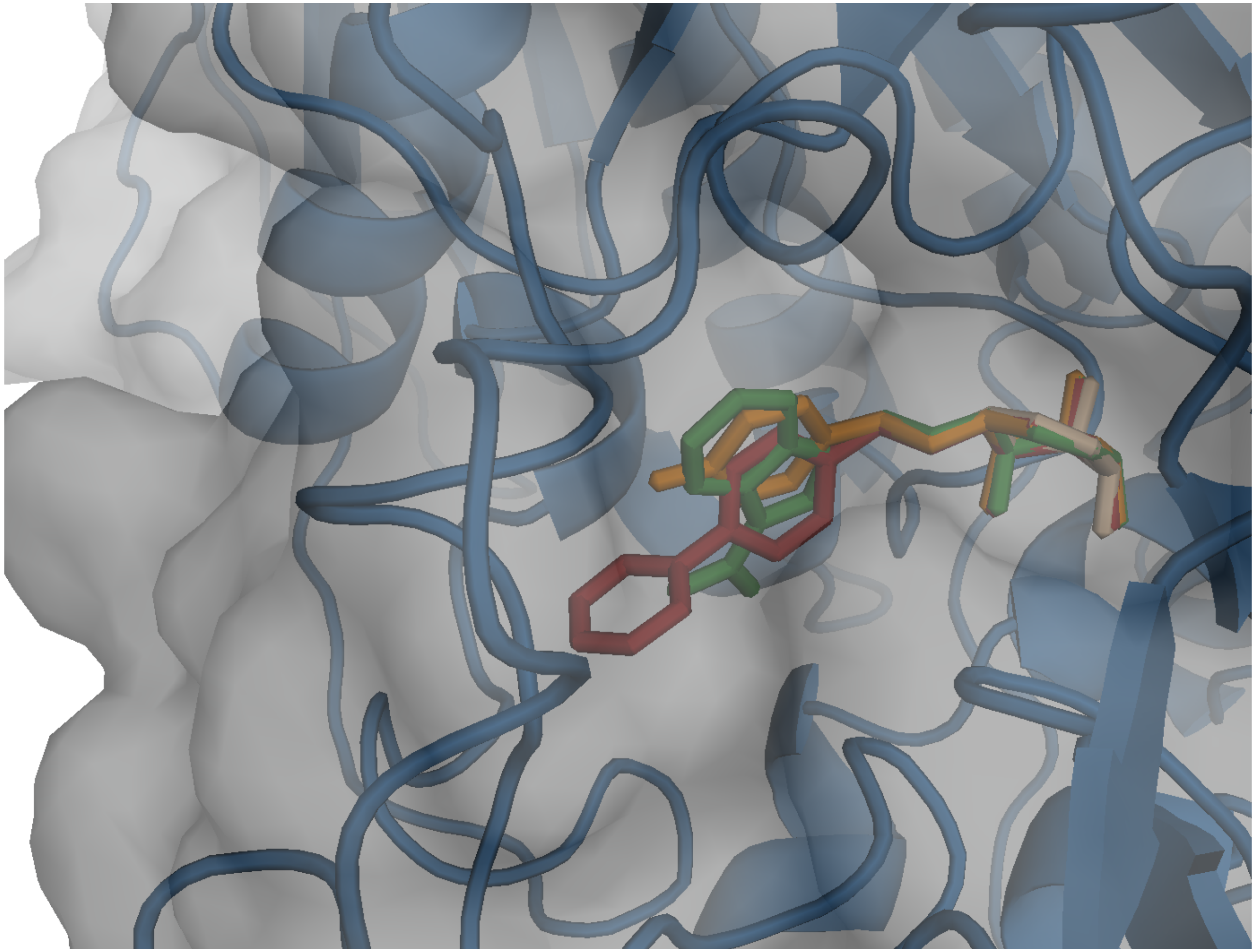

Figure S21: Superposition of the best-docked conformations of compounds **1 - 4** in the modelled α-1,4-glucosidase active site.
